# Are numerical abilities determined at early age? A brain morphology study in children and adolescents with and without developmental dyscalculia

**DOI:** 10.1016/j.dcn.2024.101369

**Published:** 2024-03-18

**Authors:** Simone Schwizer Ashkenazi, Margot Roell, Ursina McCaskey, Arnaud Cachia, Gregoire Borst, Ruth O’Gorman Tuura, Karin Kucian

**Affiliations:** a Neuropsychology, Dept. of Psychology, University of Zurich, Zurich, Switzerland; b Center for MR-Research, University Children’s Hospital Zurich, Zurich, Switzerland; c Children’s Research Center, University Children’s Hospital Zurich, Zurich, Switzerland; d Zurich Center for Integrative Human Physiology, University of Zurich, Zurich, Switzerland; e Neuroscience Center Zurich, University of Zurich and Swiss Federal Institute of Technology Zurich, Zurich, Switzerland; f Université de Paris, LaPsyDÉ, CNRS, Paris F-75005, France; g Université de Paris, Imaging biomarkers for brain development and disorders, UMR INSERM 1266, GHU Paris Psychiatrie & Neurosciences, Paris F-75005, France

**Keywords:** Numeracy, Developmental Dyscalculia, MRI, Intraparietal sulcus, Brain morphology, Children, Adolescents

## Abstract

The intraparietal sulcus (IPS) has been associated with numerical processing. A recent study reported that the IPS sulcal pattern was associated with arithmetic and symbolic number abilities in children and adults. In the present study, we evaluated the link between numerical abilities and the IPS sulcal pattern in children with Developmental Dyscalculia (DD) and typically developing children (TD), extending previous analyses considering other sulcal features and the postcentral sulcus (PoCS). First, we confirm the longitudinal sulcal pattern stability of the IPS and the PoCS. Second, we found a lower proportion of left sectioned IPS and a higher proportion of a double-horizontal IPS shape bilaterally in DD compared to TD. Third, our analyses revealed that arithmetic is the only aspect of numerical processing that is significantly related to the IPS sulcal pattern (sectioned vs not sectioned), and that this relationship is specific to the left hemisphere. And last, correlation analyses of age and arithmetic in children without a sectioned left IPS indicate that although they may have an inherent disadvantage in numerical abilities, these may improve with age. Thus, our results indicate that only the left IPS sulcal pattern is related to numerical abilities and that other factors co-determine numerical abilities.

## 1 Introduction

Mathematical competence is a highly relevant factor influencing professional and academic success (Fonteyne et al., 2015). Mathematical abilities such as arithmetic are acquired during childhood and are associated with the socio-economic status in adulthood (Ritchie & Bates, 2013). In addition, math abilities are also highly relevant for mastering daily life skills (Jansen et al., 2016). Deficits in math usually become apparent during the formal acquisition of numerical abilities in the first school years. Whereas typically developing children start solving simple problems with procedural methods (i.e., counting) and eventually develop a core knowledge of arithmetic facts that enables fast recall, children with developmental dyscalculia (DD) show impairments in the development of fact retrieval and continue to depend on procedural strategies while often using their fingers as a support (Price et al., 2013). A meta-analysis revealed that in addition to their difficulties in fact retrieval, children with DD show impairments in calculation and number sense (i.e., as expressed in quantity processing, quantity-number linking and numerical relations) and visual-spatial short-term storage (Haberstroh & Schulte-Körne, 2022). Thus, understanding brain related factors underlying numerical abilities and/or deficits are of high importance to provide knowledge needed for the development of early interventions. A key brain structure that has been associated with number processing in a range of studies is the intraparietal sulcus (IPS), recently confirmed by meta-analyses (Escobar-Magariño et al., 2022; Hawes et al., 2019). Studies with healthy and math impaired populations of adults as well as children repeatedly reported the IPS being related (functionally or structurally) to both symbolic and non-symbolic number processing (Göbel et al., 2022; Kaufmann et al., 2011; Price et al., 2007). Whereas there is substantial evidence for a pivotal role of the IPS in numeracy, the fine-tuning in understanding of how and to which extent the IPS determines numerical abilities still needs to be unraveled. An ongoing debate concerns the differences in the hemispheric contribution of the IPS to numerical abilities and findings are mixed. For example, Delazer et al. (2003) found a left-over-right IPS-dominance for complex arithmetic in adults. In contrast, Schel and Klingberg (2017) identified the right IPS to be more specifically associated with mathematical abilities, whereas the left IPS only becomes associated with mathematical abilities after the early school years, but shares the involvement in other abilities such as visual spatial working memory. Furthermore, several studies investigated laterality regarding symbolic vs non-symbolic number processing and most findings point to a left-over-right IPS-specialization for symbolic number processing (Matejko et al., 2019; Vogel at al., 2015) and a right-over-left dominance for non-symbolic number processing (Kersey & Cantlon, 2017). A lateralization index analysis of meta data (Escobar-Magariño et al., 2022) confirmed those findings. However, the results of Artemenko et al. (2020) challenge this view as they found that laterality in symbolic-number processing in the IPS is associated with handedness. For left-handed adults they found a significant right-over-left IPS-dominance during approximate symbolic calculation. For right-handers, the pattern pointed to the opposite direction, however the laterality effect was not significant. Hence, in regard of these results together with the fact that in the meta-analysis of Escobar-Magariño et al. (2022) mainly studies with right-handers were included, the proclaimed left-over-right dominance for symbolic number processing is being called into question. In other words, we cannot exclude the possibility that the reported left-over-right dominance for symbolic number processing accounts in particular for right-handers but not necessarily for left-handers. Furthermore, sub-regions of the IPS were associated with different numerical abilities (Artemenko et al., 2020, Castaldi et al., 2020) and adjacent regions to the IPS were found to be involved in number processing (Castaldi et al., 2020; Hawes et al., 2019). For example, number comparison activated a region of the inferior IPS, which extended to the postcentral sulcus (PoCS) and postcentral gyrus (Castaldi et al., 2020). Interestingly, previous studies also reported activation during number comparison in the PoCS (Chochon et al., 1999; Le Clec'H et al., 2000). In children with math disabilities or DD, differences in the activation pattern and structure of the IPS compared to typically developing children have been reported. A large number of studies found a reduced activation of the IPS in children with DD (for a review see De Smedt et al., 2019). Reduced gray matter volume in the left IPS (Isaacs et al., 2001) or in the right IPS (Cappelletti and Price, 2014, Ranpura et al., 2013, Rotzer et al., 2008) or in the bilateral IPS (Rykhlevskaia et al., 2009) was observed in children with math deficits or DD and the reduction seems to be persistent over time (McCaskey et al., 2020). Furthermore, functional connectivity analyses revealed hyperconnectivity of the IPS with several regions in the fronto-parietal cortex in children with DD, which was associated with inefficient brain processing of numerical operations (Michels et al., 2018; Rosenberg-Lee et al., 2015). Taken together, there is substantial evidence that the IPS is involved whenever there is any form of number processing. Depending on the subdomain of number processing or the format in which a number is represented, there seem to be some variations in the hemispheric contributions and in the sub-regions within and near the IPS being involved.

An open question is, to which extent deficits in numerical abilities are determined at birth or develop later in childhood due to environmental factors. These factors, including home environment, parental support, teacher competencies, math anxiety, self-esteem and socio-economic status have been reported to influence mathematical development (Kaskens et al., 2020; Muñez et al., 2021; Vukovic et al., 2013). The evaluation of this question is challenging. Theoretically, a longitudinal MR design from birth up to adulthood with contemporaneous assessment of numerical abilities and environmental factors could provide more insights into this topic. Practically, such a design is rather difficult since extensive image artefacts due to movement are expected in young children (Allievi et al., 2013). However, a promising method to address this question is the analysis of qualitative structural characteristics like the sulcal pattern (Cachia et al., 2021). The sulcal pattern is determined in utero and was observed to remain stable after term birth (Cachia et al., 2021, Chavoshnejad et al., 2021, Chi et al., 1977). This aspect stands in contrast to quantitative structural measures (e.g., sulcal depth, cortical-surface, -thickness, -volume) that undergo a significant change during development (Cachia et al., 2016) and such changes in several brain areas have also been linked with the typical development of numerical abilities in early childhood (Kuhl et al., 2020). Sulcal pattern stability in typically developed children and adults was confirmed for the anterior cingulate cortex (ACC) (Cachia et al., 2016, Tissier et al., 2018) and the inferior frontal cortex (Tissier et al., 2018). Furthermore, from birth through the age of two, the spatial distribution of sulcal pits (i.e., local maxima of depth within a sulcus) was observed to be stable between the assessed time points (Meng et al., 2014). These studies indicate that sulcal patterns are stable from term birth through adulthood and allow the assumption of IPS pattern stability, yet to our best knowledge, evidence demonstrating the stability of the IPS pattern over time is still outstanding. Moreover, to our best knowledge, sulcal pattern stability has only been investigated in typically developed individuals. While it is highly likely that sulcal pattern are also stable in atypical populations, including individuals with DD, this has not yet been evaluated. A further characteristic of sulcal patterns is its high qualitative variability (e.g., presence/absence, interruption/continuation, spatial organization) among individuals (Cachia et al., 2021, Mellerio et al., 2016). Consequently, because of the determination in utero, the longitudinal stability that has shown in typical populations, and the individual characteristics, the evaluation of sulcal patterns may enable the identification of relationships between variations in cognitive abilities and sulcal patterns in a predictive fashion. Indeed, researchers were able to link specific sulcal patterns to certain cognitive abilities such as the sulcal pattern of the ACC with inhibitory control efficiency (Tissier et al., 2018) and the pattern of the occipito-temporal sulcus (OTS) with reading abilities in children (Cachia et al., 2018) and in adults (Borst et al., 2016). In individuals with developmental dyslexia, the sulcal basin (i.e., concave substructures of a sulcus) area in the left parieto-temporal and occipito-temporal regions correlated with reduced reading performance (Im et al., 2016). Depending on the type of the numerical ability, Roell et al. (2021) found a relationship with the sulcal pattern of the bilateral IPS in both children and adults. Specifically, they observed that the presence of perpendicular branches sectioning the IPS, as compared to their absence, was related to higher scores in arithmetic and symbolic number comparison but not to non-symbolic number comparison. In addition to this qualitative evaluation, Roell et al. (2021) also analyzed quantitative measures such as sulcal depth, cortical surface area and thickness and could not detect any relationship with task performance. These results support the specificity of their finding that the sulcal pattern of the IPS (i.e., sectioned vs not sectioned IPS) is related to numerical abilities.

The aims of the present study were multifold. First, we aimed to assess the sulcal pattern stability of the IPS and the PoCS by evaluating longitudinal brain data from children with DD and typically developing (TD) children. Second, we enlarged the number of criteria of sulcal pattern features to provide a more conclusive picture of the IPS and its close neighborhood. In this criteria set we also included the PoCS based on previous studies that showed associations between numerical abilities and activation in the PoCS (Castaldi et al., 2020, Chochon et al., 1999; Le Clec'H et al., 2000). Moreover, based on previous reports showing variations in the IPS shape in individuals with Turner syndrome, a genetic syndrome associated with the development of DD (Molko et al., 2003), we also defined a shape criterion describing empirically seven different shapes of the IPS. Third, we evaluated whether TD children and children with DD differ in their sulcal pattern. Considering the finding of Roell et al. (2021), we expected that the large majority of children with DD would have a non-sectioned IPS bilaterally and compared to TD children show a significantly lower incidence of sectioned IPS with a medium-large effect. Furthermore to evaluate the specificity of the sectioning feature we also tested whether the groups differed in the amount of branches connected to the IPS regardless of whether it was a section or not. We based this criterion on the consideration that a higher number of sulcal branches also reflects a higher number of gyri, and on the finding that white matter fiber density differs between gyri and sulci such as that gyri compared to sulci have a higher density of fiber connections (Chavoshnejad et al., 2021, Li et al., 2015). Thus, it is possible that white matter architecture in terms of fiber density rather than the sectioning itself is related to numeracy. Since the outcome of Roell et al. (2021) demonstrated the specificity of the sectioning feature by applying quantitative analyses, we did not expect the groups to differ significantly in the number of IPS connected branches. For the feature whether the IPS is interrupted or continuous we expected the children with DD to show a significantly higher rate of interrupted IPS, based on previous reports in literature describing the presence of interrupted IPS in individuals with Turner syndrome (Molko et al., 2003). Furthermore, based on reported higher activation of the PoCS for number comparison (Castaldi et al., 2020; Chochon et al., 1999; Le Clec'H et al., 2000) we expected the pattern of the PoCS and its relations with the IPS to significantly differ between the groups. Fourth, we aimed to replicate the findings of Roell et al. (2021) and therefore expected that the presence of a sectioned IPS in both hemispheres is significantly related to higher symbolic number comparison and arithmetic (i.e., addition, subtraction, multiplication) but not to non-symbolic number comparison, showing a medium to large effect. Moreover, we expected that addition and subtraction will be stronger related to the sulcal pattern compared to multiplication. We based this assumption on reports showing that solving strategies do differ among those arithmetic tasks such as that multiplication relies more on fact retrieval and addition and subtraction more on calculation (Dehaene et al., 2003, Fayol and Thevenot, 2012) Furthermore, stronger activations of the IPS for subtraction compared to multiplication have been reported (Chochon et al., 1999; Prado et al., 2011). Finally, in an explorative approach we investigated whether numerical abilities other than those tested in the study of Roell et al. (2021) are related to the sulcal pattern.

## 2 Method

### 2.1 Study design and participants

We defined three different sub-studies with five study groups in total. All data derived from existing data sets of studies previously conducted at our center. For the analysis of longitudinal sulcal stability (sub-study 1) we included 34 participants of a longitudinal study (McCaskey et al., 2018). Due to insufficient MR data quality (see 2.3.2) we had to exclude two participants, which resulted in a final sample of 14 TD and 18 DD. Children that had participated in this study were tested at two time points, on average 4.1 years apart. To evaluate possible group differences (i.e., DD vs TD) in the sulcal pattern of the PoCS, the IPS and its connection to neighboring sulci (sub-study 2) we included 88 participants from four different studies (Kucian et al., 2011, Kucian et al., 2018, McCaskey et al., 2017, Rotzer et al., 2008). After the exclusion of two participants with insufficient data quality (see 2.3.2) our final sample was composed of 44 DD and 42 TD. To evaluate the relation of numerical abilities and sulcal pattern feature #1 (i.e., sectioned vs non-sectioned IPS) (sub-study 3) we included in total 68 participants, who derived from the same studies as our sub-study 2 except for McCaskey et al. (2017) since these participants were not assessed using the Neuropsychological Test Battery for Number Processing and Calculation in Children – Revised (ZAREKI-R) and were therefore not included. See Table 1 for an overview of the sub-studies, study groups and descriptive information.

**Table 1:** Study designs and study groups.

*Sub-study*	1) Longitudinal sulcal stability				2) Sulcal pattern DD vs TD		3) Association of numerical abilities and sulcal pattern
	**Time 1**		**Time 2**				
*Diagnosis*	ZAREKI-R		Basis-Math		ZAREKI-R and Basis-Math		ZAREKI-R
*Group*	DD	TD	DD	TD	DD	TD	children (continuity approach)
*n*	18^a^	14^a^	18^a^	14^a^	44^a^	42^b^	68^a^ (34 DD, 34 TD)
*Age*	M 9.7, SD 0.9, range 8.2–11.6	M 9.4, SD 1.1, range 7.9–11.3	M 13.7, SD 1.2, range 12.1–16.5	M 13.7, SD 1.1, range 12.3–16.1	M 10.1, SD 1.8, range 7.8–16.5	M 10.2, SD 1.9, range 7.9–16.5	M 9.7, SD 1.1 range 8–12 (DD: M 9.8, SD 1.1 range 7.8–11.7, TD: M 9.6, SD 1.1 range 7.9–11.4)
*Gender*	14 f	7 f	14 f	7 f	34 f	30 f	49 f (DD: 26 f, TD: 23 f)
*Handedness*	11 R, 2 L, 5 A	8 R, 1 L, 5 A	11 R, 2 L, 5 A	8 R, 1 L, 5 A	34 R, 2 L, 8 A	33 R, 3 L, 8 A	51 R, 5 L, 12 A (DD: 26 R, 2 L, 6 A, TD 25 R, 3 L, 6 A)
*IQ measures*	WISC-III		WISC-IV		WISC-III/IV		WISC-III/IV
*Estimated IQ*	102.4 (SD 6.0)	111.7 (SD 6.7)	101.5 (SD 6.4)	111.8 (SD 6.2)	99.5 (SD 6.8)	109.9 (SD 7.0)	104.3 (SD 9.2) (DD: 98.9 (SD 7.5), TD: 109.9 (SD 7.3)

Note: DD = children with developmental dyscalculia, TD = typical developed children, n = number of participants included in the analyses, M = mean, SD = standard deviation, f = female, R = right handed, L = left handed, A = ambidextrous, ^a^due to lateral MRI artifact one left and three right hemispheres excluded, ^b^due to lateral MRI artifact one right hemisphere excluded.

Power analyses for sub-study 2 and sub-study 3 are displayed in Supplementary Chapter 2. Only participants that had been included in the previous studies based on their behavioral performance were included in our sub-studies. These inclusion criteria were that all participants had an IQ above 85 and no history of neurologic or psychiatric disorder. In all of our three sub-studies the children with DD had a significant lower estimated IQ compared to TD children (sub-study 1: time-point 1: *t*(30) = −3.98, *p* < .001, Cohen's *d* = 1.46, time-point 2: *t*(30) = −4.55, *p* < .001, Cohen's *d* = 1.63*;* sub-study 2: *t*(83) = −6.93, *p* < .001, Cohen's *d* = 1.51; sub-study 3: *t*(66) = −6.05, *p* < .001, Cohen's *d* = 1.49). All typically developed children demonstrated age-appropriate mathematical performance. The diagnosis of DD was made based on the diagnostic values of standardized numerical batteries. Depending on the study, the diagnostic criteria were met if either the total score or the score on three subtests were below the 10th percentile of the ZAREKI-R or if the basic knowledge was not reached in the BASIS MATH (Moser Opitz, 2010; see McCaskey et al., 2018). Age and gender did not differ significantly between the groups (age: sub-study 1: time-point 1: *t*(30) = 0.809, *p* = .425, Cohen's *d* = 0.30, time-point 2: *t*(30) = −0.037, *p* = .971, Cohen's *d* = 0; sub-study 2: *t*(84) = −0.227, *p* = .821, Cohen's *d* = 0.05; sub-study 3: *t*(66) = 0.869, *p* = .388, Cohen's *d* = 0.18, gender: sub-study 1: (time point 1 = time point 2) X^2^ (1, *N* = 32) = 1.561, *p* = .212, Phi (φ) = 0.22; sub-study 2: X^2^ (1, *N* = 86) = 0.386, *p* = .535, φ = 0.07; sub-study 3: X^2^ (1, *N* = 68) = 0.657, *p* = .417, φ = 0.10). Informed and written consent was available from all children's caregivers and from participants older than 16 years. The study was approved by the Ethics committee of Zurich, Switzerland based on guidelines from the World Medical Association’s Declaration of Helsinki (WMA, 2013).

### 2.2 Behavioral measures

For each participant, handedness, numerical abilities and IQ were assessed using age-appropriate neuropsychological tests as described in the following section.

#### 2.2.1 Handedness

Handedness was determined by the Edinburgh Handedness Inventory (Oldfield, 1971). This inventory contains questions about or requests of performing activities of daily living, which are scored and based upon resulting in a laterality quotient of either left-handedness, ambidexterity or right-handedness.

#### 2.2.2 Numerical abilities

Numerical abilities were assessed using the ZAREKI-R (von Aster et al., 2006). This test battery contains 12 subtests assessing a range of distinct basic numerical abilities (see Supplementary Table 1). For our third sub-study (i.e., evaluation of relation between the sulcal pattern and numerical abilities) we used the three subtests addition, subtraction and multiplication as analogous to the arithmetic test used by Roell et al. (2021). The subtest perceptual estimation of quantity we used to reflect non-symbolic number ability and the subtest number comparison to reflect symbolic number ability. These tasks differed in some aspects from the tasks used by Roell et al. (2021) as our tasks were not time constrained and instead of symbolic and non-symbolic comparison of a number range from 1 to 9 (Roell et al., 2021) our participants had to compare multi-digit numbers and to estimate non-symbolic quantity. The remaining subtests (i.e., enumeration, counting backwards, writing numbers, reading numbers, number line, digit span, oral number comparison, contextual quantity estimation, story problems) were used for the exploratory analyses.

#### 2.2.3 Intelligence quotient

Depending on the original study in which the children participated, intelligence was measured with the third, or fourth edition of the Wechsler Intelligence Scale for Children (WISC) (Petermann and Petermann, 2007, Tewes et al., 1999) using a selection of the subtests (WISC-III: Similarities, Block Design, Vocabulary, Picture Arrangement; WISC-IV: Similarities, Block Design, Matrix Reasoning).

### 2.3 Neuroimaging

#### 2.3.1 Image acquisition

The high-resolution three-dimensional anatomical images of the described data sets had been acquired on a 3 T General Electric Discovery 750 Magnetic Resonance Imaging scanner (GE Medical Systems, USA) using an 8-channel head coil. Images were obtained parallel to the anterior–posterior commissure line with a T1-weighted structural image using a fast spoiled gradient echo sequence (3D FSPGR). Depending the source of the data the sequence parameters slightly differed. Twenty-eight images of sub-study 1, 49 images of sub-study 2, and 39 images of sub-study 3 were obtained using a 3D FSPGR with the following sequence parameters: number of slices = 172, slice thickness = 1 mm, no interslice skip, matrix size = 256 × 256, field of view = 240 × 240 mm, voxel size 0.93 × 0.93 × 1.00 mm, FA = 20°, TE = 3 ms, TR = 10 ms, TI = 500 ms. The remaining images were obtained using a 3D FSPGR with the following sequence parameters: number of slices = 172, slice thickness = 1 mm, no interslice skip, matrix size = 256 × 256, field of view = 256 × 256 mm, voxel size 1 mm^3^, FA = 8°, TE = 3 ms, TR = 10 ms, TI = 600 ms.

#### 2.3.2 Image quality test and processing

All images had gone through quality check in their original study. In addition, we assessed the image quality using the IQR (image quality rating) score of the Computational Anatomy Toolbox CAT12 software (Gaser et al., 2022). All images had a sufficient score of their data quality, see Supplementary Table 2.

In order to examine the sulcal patterns we processed the images using the “Morphologist toolbox” of the BrainVISA 4.6.0 software with standard parameters (https://brainvisa.info/). An automated pre-processing step was performed to skull-strip the T1 MRIs, to segment the brain tissues, to separate the two hemispheres and to reconstruct the 3D-surfaces. No spatial normalization to a common space was applied in order to avoid potential bias resulting from sulcal shape deformations induced by the warping process. The automatic segmentation of the cortical folds throughout the cortex was based on the skeleton of the gray matter/cerebrospinal fluid mask (Mangin et al., 2004). This procedure has been shown to yield a stable and robust sulcal surface definition that is not affected by variations in the gray-matter/white-matter contrast or in cortical thickness due for instance to slight differences in voxel size. One participant was excluded due to a failure in the image processing. Furthermore, at each processing step images were visually inspected for irregularities. Based upon these visual inspections, in one participant the left hemisphere was excluded, in three participants the right hemisphere was excluded and four participants were totally excluded.

#### 2.3.3 Classification of sulci

##### 2.3.3.1 Classification procedure

The image processing provided individual three-dimensional meshes of the cortical folds for manual labelling based on visual inspection (see 2.3.3.2). In a second step, the sulcal patterns were rated according to given criteria of morphological features (see 2.3.3.3). All data sets were anonymized and did not contain any behavioral data. In order to avoid possible influence of available sulcal information the manual labelling and rating of the sulci of interests were conducted blind between the left and right hemisphere. This procedure was carried out independently by two experts. The average interrater reliability across the sulcal pattern feature #1 - #6 was 94.6% and the interrater reliability of the feature #7 was 88.4%. All cases of whom the experts had a different outcome were discussed by the experts to reach consensus.

##### 2.3.3.2 Sulci identification and labelling

**Fig. 1:**
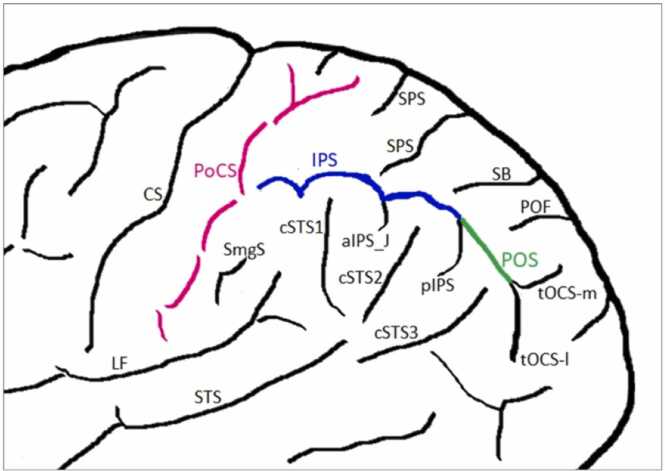
Labelled sulci: central sulcus (CS), postcentral sulcus (PoCS), longitudinal fissure (LF), supramarginal sulcus (SmgS), superior temporal sulcus (STS), caudal superior temporal sulcus (cSTS) 1, 2 and 3, intraparietal sulcus (IPS), anterior intermediate parietal sulcus (Jensen) (aIPS_J), posterior intermediate parietal sulcus (pIPS), superior parietal sulci (SPS), sulcus of Brissaud (SB), posterior occipital fissure (POF), paroccipital sulcus (POS), transverse occipital sulcus media (tOCS-m) and transverse occipital sulcus lateral (tOCS-l).

Following the atlas of Petrides (2018) all critical sulci needed to accurately identify the sulci of interest were labeled (as illustrated in Fig. 1).

The IPS was labelled by applying the criteria of Zlatkina and Petrides (2014). Therefore, posteriorly we labelled the IPS as far as the sulcus of Brissaud (SB) and the anterior part of the paroccipital sulcus (POS) which was identical to the criteria used in the study of Roell et al. (2021). Anteriorly, we labelled the IPS after initially identifying the central (CS) and PoCS, the supramarginal sulcus (SmgS) and caudal superior temporal sulcus 1 (cSTS1). Furthermore, as an orientation help we used the four IPS types identified by Zlatkina and Pierides (2014).

##### 2.3.3.3 Sulcal pattern features

**Fig. 2:**
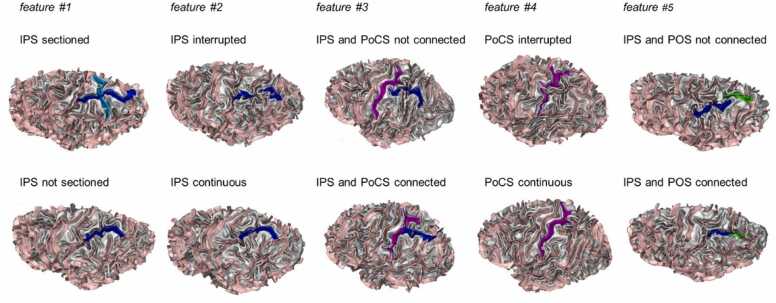
The five two-level sulcal pattern features: #1 intraparietal sulcus (IPS) sectioned vs not sectioned, #2 IPS interrupted vs continuous, #3 IPS connected vs not connected with the post central sulcus (PoCS), #4 PoCS interrupted vs continuous, and #5 IPS connected vs not connected with the paroccipital sulcus (POS).

**Fig. 3:**
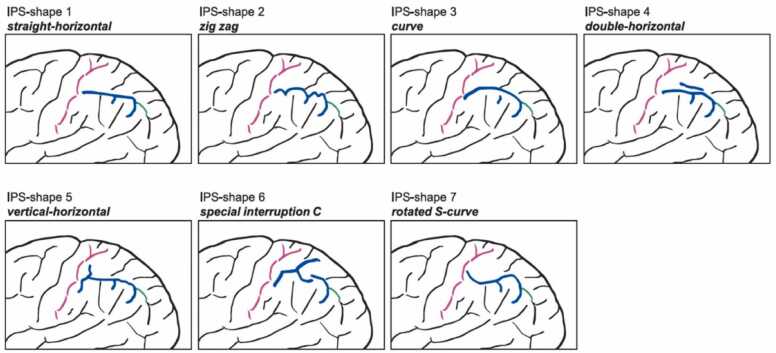
IPS-shape feature #7. IPS illustrated in blue color. Shape 1 is characterized by a straight-horizontal shape without any windings, shape 2 describes a zigzag or snake shape in a horizontal direction, shape 3 describes a single curve without any windings, shape 4 double-horizontal is defined by a second sulcus in horizontal parallel direction to the main IPS sulcus, shape 5 is characterized by a vertical part and a horizontal part which can be connected or separated from each other, shape 6 is characterized by an interrupted IPS in the form of a prominent C (inversed C in right hemisphere), shape 7 describes the shape of a S-curve rotated 90° to the left.

We defined to rate in total seven sulcal pattern features. The following five features had two levels: **#1** IPS sectioned vs not sectioned, **#2** IPS interrupted vs continuous, **#3** IPS connected vs not connected with the PoCS, **#4** PoCs interrupted vs continuous, and **#5** IPS connected vs not connected with the POS, see Fig. 2**.** Feature **#6** was continuous and described the number of branches connected to the IPS (regardless of whether it was sectioned, if there was a sectioning it was counted as two branches) and the last feature **#7** described seven different shapes of the entire IPS as a whole, see Fig. 3**.**

The first sulcal pattern feature (IPS sectioned vs not sectioned) was identical to the one used in the study of Roell et al. (2021) such as that a presence of a sulcus totally sectioning the IPS was classified as sectioned and an absence of such a sulcus was classified as 'not sectioned'. The second feature (IPS interrupted vs continuous) we based on the study of Zlatkina and Petrides (2014) that demonstrated that the IPS can be continuous or divided into two or three segments. The third feature (IPS and PoCS connected vs not connected) was based on previous reports describing the PoCS to be merged with the IPS or separated from it by a narrow gyrus (Ono et al., 1990; Zlatkina & Petrides, 2010). The fourth feature (PoCS interrupted vs continuous) refers to studies done by Zlatkina and colleagues (Zlatkina et al., 2016, Zlatkina et al., 2022) who described the PoCS as a complex of usually five distinct sulcal segments, which become evident in deeper sulcal layers. On the surface, some or even all of these segments may be merged. We therefore defined the feature of whether the PoCS is continuous or interrupted at one or more of the described segment-locations. The fifth feature (IPS and POS connected vs not connected) is based on the finding of Zlatkina and Petrides (2014) that when the inspection was done from the surface, the posterior end of the IPS was separated from the POS in half of their cases in the left hemisphere and in 65% of their cases in the right hemisphere. For the sixth feature (number of branches connected with the IPS) we counted the number of connected branches regardless of whether this was a sectioning or not (i.e., a sectioning was counted as two connected branches). This feature (see introduction) we defined in addition to its qualitative expression in order to evaluate the specificity of the first feature (IPS sectioning). The seventh and last feature described the overall shape of the IPS. Based on empirical evaluation of our data, we identified seven different shapes, defined and illustrated in Fig. 3. In all included participants apart from the left IPS in one child (with DD), we could classify the IPS according to these shapes.

### 2.4 Statistical analyses

All statistical analyses were conducted using IBM SPSS Statistics (Version 26) predictive analyses software.

#### 2.4.1 Sub-study 1: longitudinal sulcal stability

In order to evaluate sulcal stability we compared the morphological data (i.e., sulcal pattern features #1 - #6) of sub-study 1 which was composed of participants that underwent an MRI scan at two time points, of about 4.1 years apart in average (see Table 1). We calculated a differential score based on absolute number counts per single sulcal pattern feature, hemisphere and group. The descriptive data was interpreted, and no statistical analyses were performed.

#### 2.4.2 Sub-study 2: distribution of sulcal patterns within and between groups

In order to evaluate possible differences of the proportions of the sulcal pattern features #1 - #5 and #7 between the groups (sub-study 2, see Table 1) we used Chi Square test or the Fisher's Exact test if the expected count in a cell was less than five. The same tests we used to evaluate whether the proportions differed between the left and right hemisphere within the groups. Variables of features #1 - #5 all had two levels (sulcal pattern feature: present vs absent) and feature #7 had seven levels (seven different shapes). In addition, using Fisher's Exact test, we also evaluated whether the distribution between the shapes (feature #7) per group was even or rather more or less frequent. For the continuous feature #6 'number of branches' we used independent t tests to evaluate possible group differences and a paired-samples t test to test for possible differences between left and right hemispheres per group.

#### 2.4.3 Sub-study 3: relationship between numerical abilities and sulcal pattern

##### 2.4.3.1 Arithmetic, symbolic and non-symbolic number comparison and sulcal pattern

Our hypotheses concerning the relationship between sulcal pattern and number abilities as outlined in the introduction were specific for each of the numerical abilities such as addition, subtraction, multiplication, symbolic and non-symbolic number comparison. Therefore, we evaluated in our participants (n = 68) whether a left or right sectioned vs not sectioned IPS (i.e., feature #1) is associated with participant’s accuracy for those numerical abilities. For each numerical ability we conducted a separate analysis of covariance (ANCOVA) correcting for age, or analysis of variance (ANOVA) if the assumptions for ANCOVA were not met. We defined the relevant ZAREKI-R subtest (i.e., accuracy (raw data) in percentage) as the dependent variable, the right IPS sulcal pattern ('sectioned' vs 'not sectioned') and the left IPS sulcal pattern ('sectioned' vs 'not sectioned' as categorical fixed factors and age as a continuous covariate. These analyses were planned a priori by following a continuous approach, without the categorical division of group (DD vs TD) since we were interested in exploring the relation between numerical abilities and the IPS pattern independent of a diagnosis of DD.

Prior to statistical hypothesis testing, we tested whether our data fulfilled the assumptions for ANCOVA. Age significantly correlated with symbolic number comparison (*r*(66) = .39, *p* = .001) and the three arithmetic subtests, addition (*r*(66) = .40, *p* < .001), subtraction (*r*(66) = .29, *p* = .015), multiplication (*r*(66) = .46, *p* < .001) and therefore confirmed the linear relationship between the dependent variables and the covariate. However, age was not significantly correlated with non-symbolic quantity perception (*r*(66) = −.07, *p* = .562. The assumption of homogeneity of the regression slopes (i.e., age x left IPS sulcal pattern, age x right IPS sulcal pattern) was confirmed for symbolic number comparison and subtraction but violated for addition (significant interaction of age and left IPS sulcal pattern, *F*(1, 58) = 11.93, *p* < .001, ηp² = .171), and multiplication (significant interaction of age and left IPS sulcal pattern, *F*(1, 58) = 9.77, *p* = .003, ηp² = .024). Consequently, for the analyses with the dependent variables addition, multiplication and non-symbolic number comparison, we excluded the covariate age and thus conducted ANOVA analyses with the factors described previously. Our dependent variables were not normally distributed (see Supplementary Fig. 1) however, literature has shown that AN(C)OVA is robust against this violation (Blanca Mena et al., 2017) as long as the shapes of the two groups (i.e., sectioned vs not-sectioned IPS) are similar. The distributions of our fixed factors were comparable with in total 31 participants with a sectioned left IPS and 36 participants with a non-sectioned left IPS and 30 participants with a sectioned right IPS and 35 participants with a non-sectioned right IPS.

##### 2.4.3.2 Diverse numerical abilities and sulcal pattern (exploratory analyses)

For the exploratory analyses evaluating whether other numerical abilities other than those analyzed a priori, we analyzed in the participants of the sub-study 3 (n = 68) the remaining assessed numerical abilities (i.e., ZAREKI-R subtests no. 1, 2, 3, 5, 6, 7, 8, 10, 11 – see Supplementary Table 1) by following the same statistical procedure as described above. The assumption of a significant correlation between age and the dependent variable was confirmed for ZAREKI-R subtests counting backwards, writing numbers, reading numbers and story problems but not for enumeration, number line, digit span, oral number comparison and contextual quantity estimation, see Supplementary Table 4. The assumption of homogeneity of regression slopes was violated for ZAREKI-R subtest story problems (significant interaction of age and left IPS sulcal pattern, *F*(1,58) = 4.99, *p* = .030, ηp² = .079). For subtests fulfilling the assumptions we conducted ANCOVAs and for the subtests that violated the assumptions we conducted ANOVAs, with the same models as described for the a priori analyses.

#### 2.4.4 Post-hoc analyses

##### 2.4.4.1 Sub-study 2: IQ and IPS sulcal pattern

Due to the fact that the DD groups demonstrated significant lower IQ compared to the TD groups, we evaluated in a post hoc analysis whether IQ was significantly related to the sulcal pattern. We conducted a Spearman correlation analysis between the IQ score and the binary score of the sulcal pattern (sectioned vs not sectioned IPS) of the entire sample of sub-study 2 (n = 86).

##### 2.4.4.2 Arithmetic in DD subgroups of IPS sulcal pattern

Considering the outcome of the group differences in the sulcal pattern feature #1 (i.e., sectioned vs not sectioned IPS) and feature #7 (IPS-shape) (see 2.3.2, 3.2) we aimed to evaluate whether the group of DD participants that showed the combination of a non-sectioned IPS and presence of the IPS shape double-horizontal differ in their numerical abilities from children with DD that had a non-sectioned IPS and a different IPS shape. Therefore, we performed independent t tests comparing these two sub-groups in their arithmetic scores. We chose these scores based on our finding that only the arithmetic subtests were significantly related to the sulcal pattern feature #1 (see 2.3.3.1, 3.3.2). Finally, we also conducted the same analyses for the DD subgroups only related to their IPS-shape such as whether they had a shape 4 double-horizontal or another shape.

##### 2.4.4.3 Effect of relationship of age and arithmetic on IPS sulcal pattern

Based on the outcome of the testing for homogeneity of the regression slopes within sub-study 3, that revealed inhomogeneity of the regression slopes only with the left but not right IPS sulcal pattern (see 2.4.3.1) and the results under 3.3.1, we wanted to further investigate the effect of the relationship of age and arithmetic performance on the IPS sulcal pattern. Therefore, we conducted post-hoc analyses of Kendall's tau correlations between age and each of the arithmetic tests (i.e., accuracy (raw value) in percentage) separately for the group of participants with a left sectioned IPS (n = 31) and for the group of participants without a left sectioned IPS (n = 36).

## 3 Results

### 3.1 Sub-study 1: longitudinal sulcal stability

Sulcal stability was evaluated by comparing the absolute numbers of sulcal pattern feature #1 - #6 of totally 32 participants (i.e., DD: n = 18, TD: n = 14) that underwent a MRI scan at two time points (in average 4.1 years apart). These comparisons revealed in both hemispheres no differences in the number of sectioned IPS and not sectioned IPS respectively, between the two time points. For the remaining features, we observed minor differences of maximum ±2 for the features #2 and #4 and max. ±1 for the features #3, #5 and #6. The absolute numbers of the sulcal pattern features #1 - #6 per group, hemisphere and time point as well as the differential scores are displayed in Supplementary Table 3.

### 3.2 Sub-study 2: distributions sulcal patterns within and between groups

**Fig. 4:**
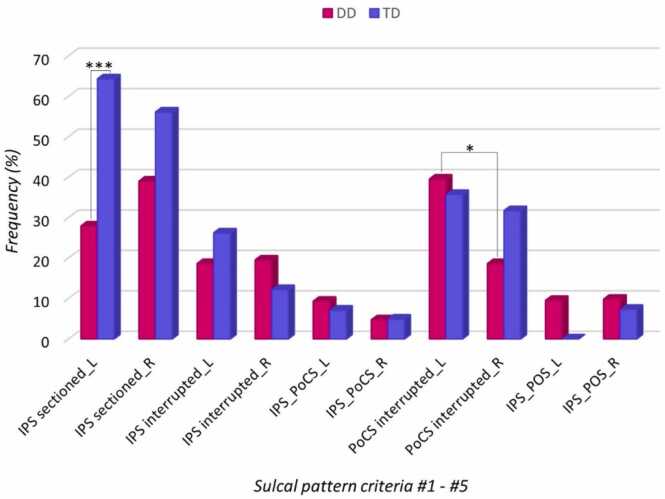
Group differences within sub-study 2 (DD = children with Developmental Dyscalculia, n = 44, TD = typically developing children, n = 42) in the proportions of sulcal pattern features #1 - #5 in left (L) and right (R) hemisphere. * = *p* < 0.05. *** = *p* < 0.001.

As can be seen in Fig. 4, in both hemispheres, children with DD (n = 44) demonstrated a lower proportion of IPS that were sectioned (i.e., feature #1) compared to the TD group (n = 42), see Table 1 for further demographic information of the groups This difference was significant only in the left hemisphere but not in the right hemisphere, and the proportion of 'sectioned' vs 'not sectioned' did not significantly differ between left and right IPS. The proportions of the features #2 - #5 did not differ significantly between the groups. Significant differences in left vs right hemisphere proportions were observed in DD in feature #4 (PoCS interrupted vs continuous) such as that the proportion of interrupted PoCS was significantly higher in the left hemisphere. In the remaining features the proportions within the groups between left and right hemisphere did not differ significantly. See Table 2 for the observed frequencies and the statistical tests values of features #1 - #5.

**Table 2:** Descriptive Statistics and Chi Square/Fisher Exact Test Results comparing between-group differences in left and right hemisphere and within-group laterality of features #1 - #5.

	Left Hemisphere					Right Hemisphere						
	A	B				A	B					
	**n (% w/i)**	**n (% w/i)**	**T**	***p*****(G)**	**X**^**2**^	**n (% w/i)**	**n (% w/i)**	**T**	***p*****(G)**	**X**^**2**^	***p*****(L)**	**X**^**2**^**(L)**
	***% btw***	***% btw***			**Φ**	***% btw***	***% btw***			**Φ**		**Φ (L)**
												
*#1 IPS sectioned (A) vs not sectioned (B)*												
DD	12 (27.9)	31 (72.1)	43	.001***	11.32	16 (39.0)	25 (61.0)	41	.122	2.40	.280	1.17
	*30.8*	*67.4*			.36	*41.0*	*58.1*			.17		.12
TD	27 (64.3)	15 (35.7)	42			23 (56.1)	18 (43.9)	41			.446	0.58
	*69.2*	*32.6*				*59.0*	*41.9*					.08
T	39	46	85			39	43	82				
												
*#2 IPS interrupted (A) vs not interrupted (B)*												
DD	8 (18.6)	35 (81.4)	43	.401	0.70	8 (19.5)	33 (80.9)	41	.364	0.82	.916	0.01
	*42.1*	*53.0*			.09	*61.5*	*47.8*			.10		.01
TD	11 (26.2)	31 (73.8)	42			5 (12.2)	36 (87.8)	41			.106	2.61
	*57.9*	*47.0*				*38.5*	*52.2*					.18
T	19	66	85			13	69	82				
												
*#3 IPS and PoCS connected (A) vs not connected (B)*												
DD	4 (9.3)	39 (90.7)	43	1.0^a^		2 (4.9)	39 (95.1)	41	1.0^a^		.676^a^	
	*57.1*	*50.0*			1.33^b^	*50.0*	*50.0*			1.0^b^		2.10^b^
TD	3 (7.1)	39 (92.9)	42			2 (4.9)	39 (95.1)	41			1.0^a^	
	*42.9*	*50.0*				*50.0*	*50.0*					1.50^b^
T	7	78	85			4	78	82				
												
*#4 PoCS interrupted (A) vs continuous (B)*												
DD	17 (39.5)	26 (60.5)	43	.716	0.13	6 (18.6)	35 (81.4)	41	.067	3.36	.033*	4.57
	*53.1*	*49.1*			.04	*31.6*	*55.6*			.20		.23
TD	15 (35.7)	27 (64.3)	42			13 (31.7)	28 (68.3)	41			.699	0.15
	*46.9*	*50.9*				*68.4*	*44.4*					.04
T	32	53	85			19	63	82				
												
#*5 IPS and POS connected (A) vs not connected (B)*												
DD	4 (9.5)	38 (90.5)	42	.116^a^		4 (9.8)	37 (90.2)	41	1.0^a^		1.0^a^	
	*100*	*47.5*			^bx^	*57.1*	*49.3*			1.37^b^		0.97^b^
TD	0 (0)	42 (100)	42			3 (7.3)	38 (92.7)	41			.116^a^	
	*0*	*52.5*				*42.9*	*50.7*					^bx^
T	4	80	84			7	75	82				

Note: Comparison of proportions in percentages (%) between (btw) groups (G) of sub-study 2 (i.e., DD = children with Developmental Dyscalculia; TD: typical developed children) and differences of laterality (L) (i.e., left vs right hemisphere) within (w/i) the groups were conducted with Chi-Square Test and Phi effect sizes were computed unless otherwise noted. A and B represent the respective level of a feature as indicated in the subtitle of each feature. ^a^Fisher's Exact Test used. ^b^Odds ratio. ^bx^Odds ratio incalculable. n = observed frequency. *p* = p value. T = total observed frequency across or within groups. X^2^ = Chi-Square value. Φ = Phi effect size. * = *p* < 0.05, *** = *p* < 0.001.

To evaluate the specificity of feature #1 (i.e. sectioned vs not sectioned IPS) and at the same time a further sulcal pattern aspect, we tested whether the total number of connected branches with the IPS (feature #6) differs between the groups. The total number of branches connected with the IPS did not significantly differ between the groups either in the left hemisphere (DD: *M* = 3.8, *SD* = 1.6, TD: *M* = 4.0, *SD* = 1.0*, t*(72.9) = −0.97, *p* = .33, Cohen's *d* = 0.15) or in the right hemisphere (DD*: M* = 3.6, *SD* = 1.6, TD: *M* = 4.1, SD = 1.3, *t*(80) = −1.40, *p* = 0.17, Cohen's *d* = 0.34). Furthermore, there were no significant differences of laterality either in DD (Left*: M* = 3.9, *SD* = 1.5, Right: *M* = 3.6, *SD* = 1.6*, t*(39) = 0.74, *p* =.46, Cohen's *d* = 0.19) or in TD (Left: *M* = 4.0, *SD* = 1.0, Right: *M* = 4.1, *SD* = 1.3*, t*(40) = −0.18, *p* = .86, Cohen's *d* = 0.09).

**Fig. 5:**
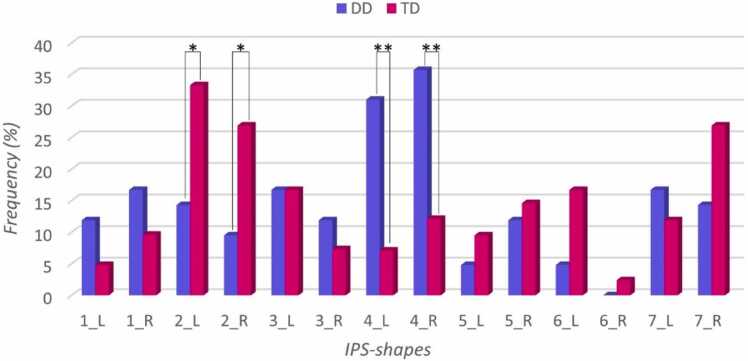
Proportions of the distribution of IPS-shapes in left (L) and right (R) hemisphere within groups of sub-study 2. DD = children with Developmental Dyscalculia; TD = typical developing children. IPS shapes 1 = straight-horizontal, 2 = zigzag, 3 = curve, 4 = double-horizontal, 5 = vertical-horizontal, 6 = special C-interruption, 7 = rotated S-curve. * = *p* < 0.05. ** = *p* < 0.01.

The distributions of IPS-shapes (feature #7) within the groups of sub-study 2 differed significantly in both groups (DD: left hemisphere, *p* = .030; right hemisphere, *p* = .039; TD: left hemisphere, *p* = .014; right hemisphere, *p* = .019) which shows that the IPS-shapes were not evenly distributed within the groups. In DD, shape 4 (residual 7.0) was most frequent in the left hemisphere and shapes 5 (residual −4.0) and 6 (residual −4.0) were least frequent in the left hemisphere. In the right hemisphere shape 4 (residual 8.0) was most frequent and shape 2 (residual −3.0) least frequent. All the remaining shapes in DD did not significantly differ in their distributions. In TD in the left hemisphere shape 2 (residual 8.0) was most frequent and shape 1 (residual −4.0) and shape 4 (residual −3.0) least frequent. In the right hemisphere, shape 6 was not present, shape 3 (residual −2.9) and shape 7 (residual −4.9) were least frequent and shape 2 (residual 5.1) and shape 7 (residual 5.1) were most frequent. The remaining IPS-shapes in TD did not differ significantly in their distributions. The investigation of the distribution of IPS shapes yielded in both hemispheres significant differences between the groups, see Table 3 and Fig. 5. The evaluation of the residuals revealed that in the left hemisphere children with DD showed a significant higher rate (*p* = .005) of shape 4 double-horizontal compared to TD children, and TD children had a significant higher rate of shape 2 zigzag (*p* = .046). Similarly, in the right hemisphere, children with DD had a significant higher rate (*p* = .012) of shape 4 double-horizontal compared to TD with a significant higher rate of shape 2 zigzag (*p* = .046).

**Table 3:** Descriptive Statistics and Fisher Exact Test Results comparing IPS-shape distribution (feature #7) in left and right hemisphere.

**IPS-shapes**	**1**	**2**	**3**	**4**	**5**	**6**	**7**	**T**	***p (group)***
	***left hemisphere***								
**DD**									
n	5	6	7	13	2	2	7	42	.021*
% w/in G	11.9	14.3	16.7	31.0	4.8	4.8	16.7		
% w/in S	71.4	30.0	50.0	81.3	33.3	22.2	58.3		
residual	1.2	-2.0*	0	2.8**	-.8	-1.8	.6		
**TD**									
n	2	14	7	3	4	7	5	42	
% w/in G	4.8	33.3	16.7	7.1	9.5	16.7	11.9		
% w/in S	28.6	70.0	50.0	18.8	66.7	77.8	41.7		
residual	-1.2	2.0*	0	-2.8**	.8	1.8	-.6		
T	7	20	14	16	6	9	12		
	***right hemisphere***								
**DD**									
n	7	4	5	15	5	0	6	42	.048*
% w/in G	16.7	9.5	11.9	35.7	11.9	0	14.3		
% w/in S	63.6	26.7	62.5	75.0	45.5	0	35.3		
residual	.9	-2.0*	.7	2.5*	-.4	-1.0	-1.4		
**TD**									
n	4	11	3	5	6	1	11	41	
% w/in G	9.6	26.9	7.3	12.1	14.6	2.4	26.9		
% w/in S	36.4	73.3	37.5	25.0	54.5	100	64.7		
residual	-.9	2.0*	-.7	-2.5**	.4	1.0	1.4		
T	11	15	8	20	11	1	17		

Note: Distributions of IPS shapes in percentages (%) within (w/in) shape (S) and group of sub-study 2 (G) (i.e., DD = children with Developmental Dyscalculia; TD: typical developed children) in left and right hemisphere were compared using the Fisher's Exact Test. n = observed frequency. T = total observed frequency across or within groups. *p* = p-value. * = *p* < 0.05. ** = *p* < 0.01.

### 3.3 Sub-study 3: relationship between numerical abilities and sulcal pattern

#### 3.3.1 Arithmetic, symbolic and non-symbolic number comparison and sulcal pattern

**Fig. 6:**
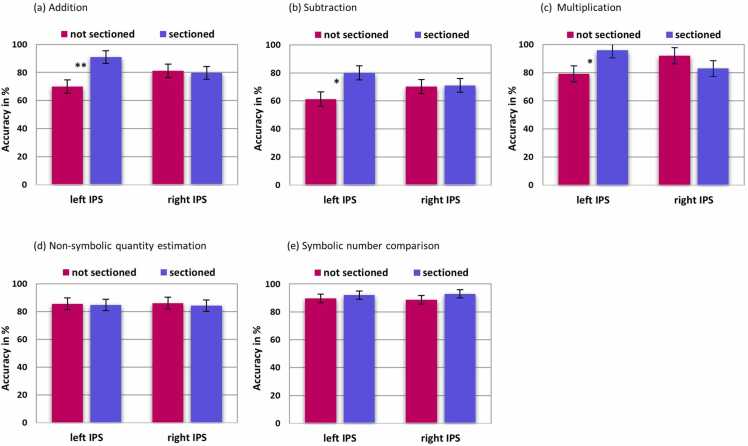
Association between (a) addition, (b) subtraction, (c) multiplication, (d) non-symbolic quantity estimation accuracy rates or (e) symbolic number comparison accuracy rates and the IPS sulcal pattern feature #1 (sectioned vs not sectioned IPS) of the continuous sample of the third sub-study (i.e., 68 children, see Table 1). Error bars depict the standard error. * = *p* < 0.05. ** = *p* < 0.01.

Univariate analyses of variance (ANOVA) and univariate analyses of covariance (ANCOVA), respectively (see Section 2.4.3.1) of totally 68 participants (see Table 1), of all three arithmetic performance indices (quantified as the percentage score relative to the maximum possible score) revealed a significant effect with the left IPS sulcal pattern feature #1. Specifically, ANOVA with dependent factor addition and the fixed factors right IPS sulcal pattern ('sectioned' vs 'not sectioned') and the left IPS sulcal pattern ('sectioned' vs 'not sectioned') showed a significant main effect of the left IPS sulcal pattern, *F*(1, 56) = 9.96, *p* = .003, ηp² = .15 (see Fig. 6a). The main effect of the right IPS sulcal pattern, *F*(1, 56) = 0.06, *p* = .813, ηp² = .001, and the interaction of the right IPS sulcal pattern and the left IPS sulcal pattern, *F*(1, 56) = 1.90, *p* = .174, ηp² = .033 were not significant.

ANCOVA with the dependent factor subtraction (see Fig. 6b) and the same fixed factors as above and the covariate age revealed that age had a significant impact on subtraction, *F*(1, 55) = 8.36, *p* = .005, ηp² = .173. The main effect of the left IPS sulcal pattern was significant, *F*(1, 55) = 6.89, *p* = .011, ηp² = .111. The main effect of the right IPS sulcal pattern, *F*(1, 55) = 0.02, *p* = .903, ηp² < .001, and the interaction of the right IPS sulcal pattern and left IPS sulcal pattern, *F*(1, 55) = 0.90, *p* = .348, ηp² = .016, were not significant.

ANOVA for the dependent factor multiplication and the same fixed factors as above revealed a significant main effect of the left IPS sulcal pattern, *F*(1, 56) = 4.54, *p* = .038, ηp² =.076 (see Fig. 6c). All remaining main effects were not significant: right IPS sulcal pattern, *F*(1, 56) = 1.33, *p* = .254, ηp² = .024, interaction of the right IPS sulcal pattern and the left IPS sulcal pattern, *F*(1, 56) = 3.45, *p* = .069, ηp² = .059. These results imply that participants with a perpendicular branch sectioning the left IPS performed significantly better in the addition, subtraction and multiplication tasks, and effect sizes indicated a medium effect for subtraction and multiplication and a large effect for addition.

The main effect of the IPS sulcal pattern was not significant, either for non-symbolic quantity estimation (see Fig. 6d) or for symbolic number comparison (see Fig. 6e). ANOVA for non-symbolic quantity estimation did not reveal any significant effects. The main effect of the right IPS sulcal pattern, *F*(1, 56) = 0.10, *p* = .759, ηp² = .002, the main effect of the left IPS sulcal pattern, *F*(1, 56) = 0.02, *p* = .89, ηp² <.001, and the interactions of the right IPS sulcal pattern and the left IPS sulcal pattern, *F*(1, 56) = 0.89, *p* = .349, ηp² = .016, were not significant.

ANCOVA for symbolic number comparison revealed that age had a significant impact on symbolic number comparison, *F*(1, 55) = 11.44, *p* = .001, ηp² = .172. The main effect of the right IPS sulcal pattern, *F*(1, 55) = 1.00, *p* = .322, ηp² = .018, the main effect of the left IPS sulcal pattern, *F*(1, 55) = 0.33, *p* = .569, ηp² = .006, and the interaction of the right IPS sulcal pattern and the left IPS sulcal pattern, *F*(1, 55) = 0.28, *p* = .600, ηp² = .005, were not significant. These results imply that participants with a perpendicular branch sectioning the IPS in either hemisphere compared to participants without a sectioned IPS did not perform significantly better in the non-symbolic quantity estimation or in the symbolic number comparison task.

#### 3.3.2 Diverse numerical abilities and sulcal pattern (exploratory analyses)

In both hemispheres of totally 68 participants (see sub-study 3, Table 1), we did not find any significant impact of the IPS sulcal pattern feature #1 (i.e., sectioned vs not sectioned IPS) on other numerical abilities such as enumeration, counting backwards, writing numbers, reading numbers, number line, digit span, oral number comparison, contextual quantity estimation or story problems. Depending on whether the assumptions for ANCOVA were confirmed or violated (see 2.4.3.1) the results of the ANCOVAs, ANOVAs respectively, are displayed in Supplementary Table 5a and 5b.

### 3.4 Post-hoc analyses

#### 3.4.1 IQ and IPS sulcal pattern

We did not detect a significant relation between IQ and the sulcal pattern of whether the IPS was sectioned or not (total sample of sub-study 2, n = 86; see Supplementary Chapter 7).

#### 3.4.2 Arithmetic in DD sulcal pattern-subgroups

To evaluate whether the subgroup of participants with DD in the third sub-study that showed the combination of a left non-sectioned IPS and the presence of a left double-horizontal IPS shape (DD non-sectioned & double-horizontal, n = 8) would show lower numerical abilities compared to DD that had a non-sectioned IPS and a different IPS shape (DD non-sectioned & different, n = 17), we performed independent t tests comparing their percentage accuracy scores for the arithmetic subtests. We chose these subtests based on our finding within sub-study 3 that showed the association of a non-sectioned left IPS with lower accuracy in addition, subtraction and multiplication. The results are displayed in Supplementary Chapter 8. We also tested by comparing the same three arithmetic tests whether the DD sub-group with a double-horizontal IPS shape (n = 9) differed from the DD sub-group with a different shape (n = 25) regardless of whether they had a sectioned or non-sectioned IPS. We found no significant differences in any of the arithmetic tests in either hemisphere (see Supplementary Chapter 9).

#### 3.4.3 IPS sulcal pattern and relationship between age and arithmetic

**Fig. 7:**
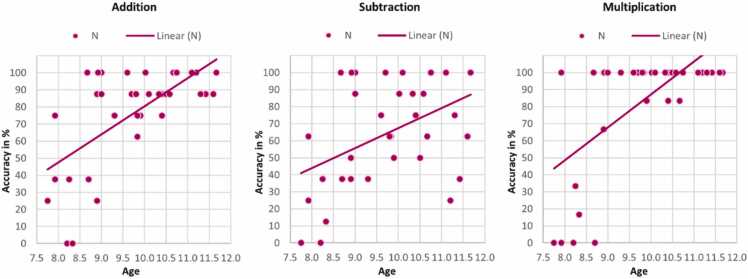
Kendall's tau correlations between age and the arithmetic accuracy scores in percentage (addition, subtraction and multiplication) for participants with a non-sectioned left IPS (n = 36) of the continuous sample of the third sub-study.

We analyzed in the participants of sub-study 3 the effect of age on the performance in all three arithmetic tests separately for participants with (n = 31) and without (n = 36) a perpendicular branch sectioning the IPS in the left hemisphere by performing two-tailed Kendall's tau correlations. We conducted this post-hoc analysis based on our findings that only the sulcal pattern of the left IPS was significantly related to addition, subtraction and multiplication and that the homogeneity of the regression slopes was violated for addition and multiplication for the left IPS (see 2.4.3.1). In the group of participants without a sectioned IPS in the left hemisphere (see Fig. 7), age was positively correlated with the performance in all three arithmetic tests: addition *r*_*τ*_(34) = .44, *p* < .001, subtraction *r*_*τ*_(34) = .30, *p* = .017, multiplication *r*_*τ*_(34) = .43, *p* = .001. These correlations did survive Bonferroni correction, with a corrected threshold of p = .017. There were no significant correlations with age in the group of participants with a sectioned left IPS: addition *r*_*τ*_(30) = −.06, *p* = .671, subtraction *r*_*τ*_(30) = .14, *p* = .326, multiplication *r*_*τ*_(30) = .16, *p* = .302. As illustrated in Supplementary Fig. 2, a large proportion of participants with a sectioned left IPS reached high arithmetic scores already at younger age, explaining the absence of a significant correlation.

## 4 Discussion

In the present study, we investigated in children and adolescents whether the sulcal pattern of the IPS and its relation with neighboring sulci and the sulcal pattern of the PoCS are stable over time (sub-study 1) and whether we can identify sulcal patterns that differentiate between children and adolescents with and without DD (sub-study 2). Furthermore, we aimed to replicate the findings of Roell et al. (2021), who demonstrated that arithmetic and symbolic number abilities, but not non-symbolic number abilities, are related to the sulcal pattern of the IPS (i.e., sectioned vs not sectioned IPS) and to investigate whether other numerical abilities are also related to the IPS sulcal pattern (sub-study 3).

As anticipated, we found that the sulcal pattern of IPS and PoCS is stable over time. We concluded this based on the observation of our descriptive data. No differences between the two time points (in average 4.1 years apart) were observed with regard to sectioned vs not sectioned IPS and only negligible differences were found for the remaining features. To our best knowledge, this is the first study to evaluate the longitudinal sulcal stability of the IPS and PoCS and the first study to evaluate sulcal stability in an atypical population such as DD. Our results confirm and extend the findings of previous studies that showed the longitudinal stability of sulcal patterns such as ACC (Cachia et al., 2016, Tissier et al., 2018) or inferior frontal sulcus (Tissier et al., 2018). Those findings, together with our observations, further support the view that sulcal patterns are stable during development (Cachia et al., 2021, Mangin et al., 2010) in both typical and atypical populations such as DD, and likely since term birth. The longitudinal sulcal stability is critical to validate the subsequent analyses aiming to link sulcal patterns with cognitive abilities, and to provide a basis for predictive statements.

Our evaluation of group differences in the sulcal pattern between children with DD and TD children (sub-study 2) confirmed our hypothesis that children with DD had a lower proportion of sectioned IPS compared to TD children. This difference was only significant in the left but not the right IPS (i.e., laterality effects will be discussed in the continuation of this paper). The observation that still about a third of the children with DD had a sectioned IPS was surprising, as we had expected a lower proportion. At the same time, this observation supports the chosen method in our third sub-study where we followed a continuity approach. Furthermore, by comparing the overall number of IPS-connected branches it is possible to provide, in addition to a further sulcal pattern feature, also an indirect quantitative statement in terms of white matter architecture (i.e., differences between gyri and sulci in fiber density connections, Chavoshnejad et al., 2021; Li et al., 2015). For instance, it is possible that the number of the connected branches, either related to qualitative (sulcal pattern) or quantitative aspects (white matter architecture) of brain morphology or to both, and not the sectioning is driving the observed group differences. Hence, our results demonstrating that the number of branches connected to the IPS did not significantly differ between the groups, supports the view that the feature of whether the IPS is sectioned or not does indeed have a special meaning for numerical abilities. Contrary to our expectations, children with DD did not show a significant higher proportion of interrupted IPS'. Our expectation was based on the finding of Molko et al. (2003) who described that individuals with the Turner syndrome linked with DD showed qualitative differences including abnormal IPS interruptions compared to typically developed individuals. It may be possible that this feature was related to other aspects of the Turner syndrome rather than pure DD. Regarding the PoCS, DD and TD did not significantly differ in their proportion of interrupted vs continuous PoCS and therefore did not confirm our expectations. However, children with DD had a significantly lower proportion of interrupted PoCS in the right compared to the left hemisphere. Whether this difference in laterality has a special meaning for DD is difficult to elaborate based on our data. Future research is necessary to evaluate whether this finding has a specific meaning related to numerical or other abilities. In the remaining sulcal pattern features we did not observe significant group differences. In both groups, the majority of the children showed a continuous IPS that was connected with the PoCS and the POS. We therefore conclude that whether the IPS is continuous or interrupted or connected or not connected with the PoCS or the POS does not have a critical meaning for numerical abilities.

When looking at the particular shape of the IPS as a whole (sub-study 2) we were able to identify seven different shapes. Two of our identified shapes resemble shapes that were previously described by Zlatkina and Petrides (2014) (i.e., shape first-vertical-then-horizontal resembles example c, p. 4, shape special interruption C-form resembles example d, p. 4). The distributions of the IPS shapes differed significantly between DD and TD. The double-horizontal IPS shape was identified in about one third of DD in both hemispheres, which was a significant higher proportion compared to that in the TD group. These results may point to a special role of the double-horizontal IPS shape for DD. However, our post-hoc analyses (sub-study 2) did not reveal clear insights into the meaning of a double-horizontal IPS shape for DD. The evaluation of whether the mutual appearance of a double-horizontal IPS shape and a non-sectioned IPS would have an additive effect leading to worse performance was not confirmed. In comparison to DD, the TD group showed a significantly higher prevalence of a zigzag IPS shape. Previous reports observed unusual IPS shapes in a sample of individuals with the Turner syndrome that is associated with DD (Molko et al., 2003). However, based on our data and outcome we cannot conclude whether the IPS shape has a specific meaning or not related to numerical abilities. Given that in both groups its dominant IPS shape is present only in about one third of each group, we assume that the IPS shape in context of numerical abilities might have a negligible meaning. Nevertheless, it is possible that the IPS shape is related to other behavioral measures not assessed in our study.

Finally, while attempting to replicate the finding of Roell et al. (2021) we could only confirm part of their results (sub-study 3). Like Roell et al. (2021), we found that higher achievements in arithmetic were significantly related to the presence of a sectioned IPS in the left hemisphere. Unlike Roell et al. (2021) we did not find this relation in the right hemisphere. Furthermore, in line with Roell et al. (2021), we could not find a relation between the IPS sectioning and non-symbolic number abilities. However, and in contrast to Roell et al. (2021), we also did not find a significant relation with symbolic number comparison. One aspect that may be related to the non-replication of this finding is that we did not use an identical task. Whereas the task of Roell et al. (2021) was composed of one-digit Arabic number pairs, the task we used (i.e., ZAREKI-R subtest no. 12) contained multi-digit number pairs. In addition, timing and response mode differed as our task was not timed and children were asked to respond by circling the larger number with a pencil, whereas in the task of Roell et al. (2021), participants were requested to answer as fast as possible by key press and had a maximum time available per trial. Thus, whereas the task of Roell et al. (2021) was more based on fluency and small Arabic numbers, our task did not involve a speed component and reflects in particular the accuracy of the knowledge related to number magnitude of complex Arabic numbers. Furthermore, we did not find significant relations of any other numerical ability (i.e., enumeration, counting, writing numbers, reading numbers, number line, digit span, contextual quantity estimation, oral number comparison, story problems) with the sulcal pattern of the IPS (i.e, sectioned or non-sectioned IPS).

Taken together, our results show that the presence of a sectioned IPS in the left hemisphere is significantly related to higher accuracy in arithmetic. None of the additionally assessed numerical abilities was significantly related to the presence or absence of a sectioned IPS. Considering that successful arithmetic is influenced by several sub-abilities such as counting and magnitude/distance representation, arithmetic fact retrieval, working memory, strategy use and others (Gruber et al., 2001), it is likely that an inherent disadvantage in number processing probably present at birth will show itself consistently in arithmetic performance. This is because deficits in each numerical ability involved in mental calculation will cumulate. Furthermore, fact retrieval and calculation abilities, both components involved in successful solving of arithmetic problems, have been identified as the abilities with which children with DD struggle most (Haberstroh & Schulte-Körne, 2022; Price et al., 2013).

The discordant finding in terms of laterality between our results (only left sectioned IPS is significantly related to DD (sub-study 2) or better arithmetic performance (sub-study 3)) and the results of Roell et al. (2021) (both, left and right sectioned IPS' were significantly related to better arithmetic performance) is challenging to explain. As it is likely that arithmetic performance is mainly based on symbolic and not non-symbolic number representation, our finding may be related to the fact that in symbolic number processing the left IPS is dominant over the right IPS (Escobar-Magariño et al., 2022). Furthermore, our results go along with the findings of Le Guen et al. (2018) who studied the genetic contributions to brain activations during a simple arithmetic task, revealing a left-hemisphere intraparietal sulcus specificity, as they found more heritable areas and higher heritability in the left IPS compared to the right IPS. However, taking into consideration that IPS laterality for number processing may be related to handedness (Artemenko et al., 2020), we cannot exclude the possibility that our results showing that only the left sulcal pattern of the IPS is related to arithmetic or to DD (i.e., lower proportion of sectioned IPS compared to TD) account in particular for right-handers but not necessarily for left-handers, as the majority of our participants were right-handed. Nonetheless, our results support the view that the IPS is differently specialized with regard to its hemispheric contribution to number processing (Le Guen et al., 2018; Vogel et al., 2015).

Finally, the question of whether numerical abilities are determined at early age we suggest to answer as follows. In our post-hoc analyses (sub-study 3) we found that for the group of participants without a left sectioned IPS, age was significantly correlated with the performance in arithmetic, such that older children showed better performance. The observation that sulcal patterns are determined in utero and stable over development, as documented in the literature (Cachia et al., 2016, Cachia et al., 2021, Chi et al., 1977, Tissier et al., 2018) and further supported by our longitudinal data including participants with DD, allows us to interpret these correlations in a directional manner. Hence, this finding indicates that although children without a sectioned IPS in the left hemisphere may have a disadvantage present at birth in the acquisition of numerical (arithmetic) abilities and a higher risk for DD, they may overcome this disadvantage or at least improve in their abilities with age. In other words, an inherent disadvantage for numerical abilities associated with the sulcal pattern is not rigidly related to DD but other developmental factors may also play a role. Our finding goes along with results reported from longitudinal studies evaluating numerical abilities in DD, which found that participants with DD showed improvement in their abilities (Landerl, 2013; McCaskey et al., 2018; Nelson & Powell, 2018) but still expressed deficits compared to typical populations. The contribution of developmental factors related to DD also becomes apparent when considering that of the children with a left sectioned IPS, which is associated with a lower risk for DD, still about one third of them were diagnosed with DD. This view complies with the current state of literature that a combination of genetic, neurobiological and environmental factors contribute to the development of DD (Klein & Knops, 2023). The main and novel finding of our study is that etiological neurobiological factors of DD are likely already present at birth, given the determination of sulcal pattern in utero (Cachia et al., 2021) and the documented sulcal pattern stability in literature (Cachia et al., 2016, Cachia et al., 2021, Mangin et al., 2010, Tissier et al., 2018) and current findings. This together with the observation that the disadvantage is likely influenced by environmental factors, emphasizes the importance of early interventions.

## 5 Limitations and future

Our goal was to identify significant relations between numerical abilities or DD and the sulcal pattern. Since we were not interested to identify which numerical abilities are unrelated to the sulcal pattern or which sulcal pattern features are not related to DD, we did not conduct equivalence tests.

Based on our data and results, we cannot give a prediction as to which degree the IPS sulcal pattern may influence numerical abilities over a lifetime. Our assumptions in terms of the relations between IPS sulcal pattern and numerical abilities are based on the test levels and age ranges assessed in this study. The absence of a significant correlation between age and arithmetic performance in the group with a sectioned left IPS is presumably related to ceiling effects, as participants already at younger age demonstrated high arithmetic scores. Therefore, it is likely that participants with a sectioned left IPS performing on arithmetic tests with a higher difficulty level would also reveal a significant relation with age. At the same time, it is possible that our finding that participants with a non-sectioned IPS showed higher arithmetic ability with age would not necessarily be observed in groups with larger age ranges and/or investigating more complex arithmetic tasks. In addition, the tasks we used were not time-constrained and therefore reflect the numerical ability, but such tasks do not differentiate between children using an efficient or less efficient strategy. Furthermore, we would like to mention that the results of our analyses concerning multiplication need to be regarded with caution due to the high rate of ceiling effects. Therefore, future research should evaluate the relation between sulcal pattern and numerical abilities in larger age-ranges, using more complex numerical problems and assessing reaction time and problem solving strategies.

In addition, the question of what the sulcal pattern feature, identified by the present study to be related to DD and arithmetic, in fact does reflect, may be further investigated. It is possible that this sulcal feature (i.e., whether the IPS is sectioned or not) reflects a genetically determined pattern common for a certain group of individuals with a predisposition for numerical abilities. Moreover, on a neurophysiological level, this sulcal pattern feature also reflects differences in white matter architecture and the way that pathways are organized, which may also affect functional brain connectivity. It therefore may be of great interest to evaluate in future studies whether participants with or without a sectioned IPS differ in functional brain connectivity.

Furthermore, we cannot entirely rule out the possibility that the second sulcus of the double horizontal IPS shape does not refer to the superior parietal sulcus, due to the fact that this second sulcus was observed dorsal to the main IPS. As mentioned above, we conclude that the IPS shape does not have a significant meaning for DD. Nevertheless, in the context of the significantly higher proportion of the double horizontal shape seen in DD compared to TD, it may be worth investigating the superior parietal sulci.

While the DD groups showed an IQ in the normal range, these groups performed significantly worse in comparison to the TD groups. This significant group difference in the IQ raises the question whether the IQ also was related to the sulcal pattern. However, one confound which arises when comparing IQ between DD and TD groups is that intelligence tests are not independent of numerical ability measures (Dennis et al., 2009). This aspect also leads to repeated reports that children with math learning disabilities differ in IQ scores from typical achieving children (Schwenk et al., 2017). Therefore, to evaluate whether IQ is related to the sulcal pattern as well, we conducted a post-hoc correlation analysis (see 2.4.4.1) between the IQ score and the binary score of the sulcal pattern (sectioned vs not sectioned IPS) of the entire sample of sub-study 2 (n = 86). The outcome of this analysis (Supplementary Chapter 7) was not significant and therefore indicates that IQ measures did not play a significant role related to the sulcal pattern.

Finally, since gender was not balanced between the groups, which also reflects the prevalence of DD affecting more females than males (Schulz et al., 2018), we could not reliably statistically test the effect of gender on the relation of sulcal pattern and numerical abilities. Therefore, we cannot provide any conclusion about gender effects on such relations.

## 6 Conclusion

In this work, we showed that the sulcal pattern of the IPS and PoCS is stable over development and that the sulcal pattern of the IPS differs between children and adolescents with and without DD. In particular, our findings indicate that the absence of a sectioned IPS has a specific meaning for numerical abilities and DD. In addition, our thorough inspection of the IPS morphology revealed a vast heterogeneity in the global shape of the IPS, as seven different shapes could be classified. Although differences in the overall IPS shape exist between DD and TD, our analyses do not support the assumption that the IPS shape has a significant implication for DD as the presence of the dominant shape (i.e., double-horizontal) was not significantly related to variations in numerical ability within DD. Finally, the analyses of the relation between IPS morphology and numerical abilities showed that the presence of a sectioned left IPS is related to better performance in addition, subtraction and multiplication. Thus, due to the presumption that the sulcal pattern of the IPS is already determined in utero, it appears to be the case that numerical abilities are partly determined at early age. This determination, however, may be limited in its meaning as indicated by the significant association between age and arithmetic performance in individuals without a sectioned IPS. Accordingly, the specific morphology of the IPS might rather be seen as an advantage or disadvantage respectively for numerical development, and other factors likely play an important role as well.

## Data statement

Data not available due to ethical restrictions.

## Funding

This research did not receive any specific grant from funding agencies in the public, commercial, or not-for-profit sectors.

## CRediT authorship contribution statement

**Simone Schwizer Ashkenazi:** Writing – review & editing, Writing – original draft, Visualization, Project administration, Methodology, Investigation, Formal analysis, Conceptualization. **Arnaud Cachia:** Writing – review & editing, Methodology. **Gregoire Borst:** Writing – review & editing. **Margot Roell:** Methodology. **Ursina McCaskey:** Writing – review & editing, Data curation. **Ruth O’Gorman Tuura:** Writing – review & editing, Supervision. **Karin Kucian:** Writing – review & editing, Supervision, Conceptualization.

## Declaration of Competing Interest

The authors declare that they have no known competing financial interests or personal relationships that could have appeared to influence the work reported in this paper.
