# Supplementary material for: Are numerical abilities determined at early age? A brain morphology study in children and adolescents with and without developmental dyscalculia
Source: Dev Cogn Neurosci. 2024 Mar 18;67:101369. doi: 10.1016/j.dcn.2024.101369 (PMC11046253; doi:10.1016/j.dcn.2024.101369)
Supplement: Supplementary file 1 — Supplementary material [file mmc1.docx]

**Supplementary Material**

**Are numerical abilities determined at early age? A brain morphology study in children and adolescents with and without developmental dyscalculia**

Simone Schwizer Ashkenazi, Margot Roell, Ursina McCaskey, Arnaud Cachia, Gregoire Borst, Ruth O'Gorman Tuura, Karin Kucian

* corresponding author: Simone Schwizer Ashkenazi ([simone.schwizer@gmail.com](mailto:simone.schwizer@gmail.com))

1. **Neuropsychological Test Battery for Number Processing and Calculation in Children – Revised (ZAREKI-R)**

The ZAREKI-R test battery is a norm-based test conceptualized to identify children with deficits in number abilities from grades 1 to 4. The ZAREKI-R consists of 12 subtests (see **Table 1**) assessing basic number skills as well as mental calculation (arithmetic). Each correct answer is scored with 1 to 2 points resulting in a total raw test score of maximum 122 points. The test has no time limit. The criteria for developmental dyslexia (DD) are considered met if the scores in three subtests belonging to the same factor fall below the 10^th^ percentile rank, or if the total test score falls below the 10th percentile rank.

**Table 1**: Description of the 12 subtests of the Neuropsychological Test Battery for Number Processing and Calculation in Children – Revised (ZAREKI-R)

| No. | Subtest | Description | Scoring | Max. Points |
| --- | --- | --- | --- | --- |
| 1 | Enumeration | Enumeration of different sets of dots (e.g. 13 dots arranged in a line, 18 dots randomly arranged) | 1 point for each correct answer | 5 |
| 2 | Counting backwards | Counting backwards starting from a given number (e.g. 22-1, 67-54) | 2 points for perfect performance, 1 point for one mistake, no points if more than one mistake | 4 |
| 3 | Writing numbers | Writing Arabic numbers from dictation (e.g. 14, 4658) | 1 point for each correct answer | 8 |
| 4a | Arithmetic Addition | Solving of mental calculations: orally presented addition problems | 1 point for each correct answer | 8 |
| 4b | Arithmetic Subtraction | Solving of mental calculations: orally presented subtraction problems | 1 point for each correct answer | 8 |
| 4c | Arithmetic Multiplication | Solving of mental calculations: orally presented multiplication problems | 1 point for each correct answer | 6 |
| No. | Subtest | Description | Scoring | Max. Points |
| 5 | Reading numbers | Reading aloud of written Arabic numbers (e.g. 305, 969) | 1 point for each correct answer | 8 |
| 6 | Number line | First part: choosing the correct position of visually or orally presented number out of four possible positions on a line from 1 to 100.  Second part: positioning of visually or orally presented numbers by marking the position on a line from 1 to 100. | first part: 1 point for each correct answer, second part: depending on the distance to the correct position the answer is worth 1 or 2 points | 18 |
| 7 | Digit span | Repeating of orally presented number sequence in the same direction (digit span forward) or in the reverse direction (digit span backward) | 1 point for each correct answer | 24 |
| 8 | Oral number comparison | Identifying the higher number out of orally presented number pairs (e.g. eight hundred – hundred and eight) | 1 point for each correct answer | 8 |
| 9 | Non-symbolic quantity estimation | Quantity estimation of visually presented sets of objects (e.g. 57 balls, 89 cups) | 1 point for each correct answer | 5 |
| 10 | Contextual quantity estimation | Quantity estimation in relation to the context if a quantity represents low, average or high quantity (e.g. fifteen words in a reading book, four fridges in a kitchen) | 1 point for each correct answer | 6 |
| 11 | Story problems | Solving of different story problems (e.g. Peter has 16 marbles. He has 4 marbles more than Anne. How many marbles does Anne have?) | 1 point for each correct answer | 6 |
| 12 | Symbolic number comparison | Identifying the larger number of visually presented Arabic number pairs (e.g. 654 or 546) | 1 point for each correct answer | 8 |

1. **Statistical power analyses**

Given our study design that used already available MRI data, we conducted an a priori power analysis using G*Power version 3.1.9.7 (Faul et al., 2007) to evaluate whether our sample sizes of sub-study 2 and sub-study 3 are large enough to test the study hypotheses. As a reference we based our expectations on the results from Roell et al. (2021) that showed a medium to large effect size of ηp² .10-.14 (corresponding to Cohen's f .33-.40, Cohen 1988) for the relation of the IPS sulcal pattern and symbolic number comparison. With a significance criterion of α = .05 and power = .80, the minimum sample size required for conducting a goodness-of-fit test (Chi-Square) in our sub-study 2, assuming a medium-to-large effect size of Cohen’s W .40, is N = 50. Similarly, for conducting AN(C)OVA in our sub-study 3, with a medium-to-large effect size of Cohen's f .35, the minimum sample size needed is N = 67. Thus, the obtained sample sizes of N = 86 for our sub-study 2 and the sample size N = 68 for our sub-study 3 are adequate to test the study hypotheses.

1. **MR Image quality test**

We computed the IQR (image quality rating) score of the software Computational Anatomy Toolbox CAT12 (Gaser, et al., 2022) for all the processed MR images that were included in our three sub-studies. This procedure revealed the following results as displayed in **Table 2**.

**Table 2**: MR image quality rating (IQR) scores and between-group statistics

|  |  | DD |  |  |  | TD |  |  | *df* | *t* | *p* value | Cohen's *d* |
| --- | --- | --- | --- | --- | --- | --- | --- | --- | --- | --- | --- | --- |
|  | *n* | *M* | *SD* | range | *n* | *M* | *SD* | range |  |  |  |  |
| Sub-Study 1 Time 1 | 18 | 80.5 | 2.5 | 74.8 – 80.5 | 14 | 80.6 | 3.1 | 70.7 – 82.9 | 30 | -.055 | .957 | 0.035 |
| Sub-Study 1 Time 2 | 18 | 80.5 | 3.2 | 69.1 – 83.9 | 14 | 79.2 | 5.1 | 63.0 – 83.0 | 30 | .835 | .410 | 0.305 |
| Sub-Study 2 | 44 | 79.9 | 3.3 | 72.1 – 86.4 | 42 | 79.7 | 3.8 | 66.0 – 84.4 | 84 | 0.269 | .788 | 0.056 |
| Sub-Study 3 | 34 | 79.7 | 3.2 | 72.1 – 85.0 | 34 | 80.1 | 3.1 | 70.5 – 84.2 | 66 | -.423 | .674 | 0.127 |

Note: Independent t tests were used to compare the image quality score (IQR) between children with Developmental Dyscalculia (DD) and typically developing children (TD).

1. **Longitudinal sulcal stability**

To investigate longitudinal sulcal stability we compared the absolute numbers of sulcal pattern features #1 - #6 (see **Table 3**) of participants included in sub-study 1. These participants underwent an MRI scan at two different time points in average 4.1 years apart.

**Table 3**. Comparison of sulcal pattern features #1 - #6 between two time points to evaluate sulcal long-term stability.

|  |  |  |  | Left Hemisphere | | | | |  | Right Hemisphere | | | | |
| --- | --- | --- | --- | --- | --- | --- | --- | --- | --- | --- | --- | --- | --- | --- |
|  |  |  |  | Time 1 | Time 2 | Change to yes | Change to no | DS |  | Time 1 | Time 2 | Change to yes | Change to no | DS |
| Sulcal pattern feature | Group | Feature present |  |  |  |  |  |  |  |  |  |  |  |  |
| #1 IPS sectioned | DD | yes / no |  | 5 /13 | 5 /13 | 0  0 | 0 | 0 |  | 6 / 10 | 6 / 10 | 0 | 0 | 0 |
|  |  |  |  |  |  |  |  |  |  |  |  |  |  |  |
|  | TD | yes / no |  | 7 / 6 | 7 / 6 | 0 | 0 | 0 |  | 8 / 5 | 8 / 5 | 0 | 0 | 0 |
|  |  |  |  |  |  |  |  |  |  |  |  |  |  |  |
| #2 IPS interrupted | DD | yes / no |  | 5 / 13 | 5 / 13 | 0 | 0 | 0 |  | 12 / 4 | 11 / 5 | 0 | 1 | 1 |
|  |  |  |  |  |  |  |  |  |  |  |  |  |  |  |
|  | TD | yes / no |  | 4 / 9 | 5 / 8 | 1 | 0 | 1 |  | 12 / 1 | 12 / 1 | 0 | 0 | 0 |
|  |  |  |  |  |  |  |  |  |  |  |  |  |  |  |
| #3 IPS-PoCS connected | DD | yes / no |  | 18 / 0 | 18 / 0 | 0 | 0 | 0 |  | 14 / 2 | 14 / 2 | 0 | 0 | 0 |
|  |  |  |  |  |  |  |  |  |  |  |  |  |  |  |
|  | TD | yes / no |  | 12 / 1 | 10 / 3 | 0 | 2 | 2 |  | 11 / 2 | 11 / 2 | 0 | 0 | 0 |
|  |  |  |  |  |  |  |  |  |  |  |  |  |  |  |
| #4 PoCS interrupted | DD | yes / no |  | 8 / 10 | 8 / 10 | 0 | 0 | 0 |  | 1 / 15 | 2 / 14 | 1 | 0 | 1 |
|  |  |  |  |  |  |  |  |  |  |  |  |  |  |  |
|  | TD | yes / no |  | 4 / 9 | 4 / 9 | 0 | 0 | 0 |  | 6 / 7 | 6 / 7 | 0 | 0 | 0 |
|  |  |  |  |  |  |  |  |  |  |  |  |  |  |  |
| #5 IPS-POS connected | DD | yes / no |  | 17 / 1 | 17 / 1 | 0 | 0 | 0 |  | 15 / 1 | 15 / 1 | 0 | 0 | 0 |
|  |  |  |  |  |  |  |  |  |  |  |  |  |  |  |
|  | TD | yes / no |  | 13 / 0 | 13 / 0 | 0 | 0 | 0 |  | 12 / 1 | 13 / 0 | 1 | 0 | 1 |
|  |  |  |  |  |  |  |  |  |  |  |  |  |  |  |
| #6 Number connected Branches | DD |  |  | 65 | 63 | 0 | 2 | 2 |  | 59 | 59 | 1 | 1 | 2 |
|  | TD |  |  | 48 | 46 | 0 | 2 | 2 |  | 54 | 53 | 0 | 1 | 1 |

Note: Comparison of absolute numbers of presence (Yes) or absence (No) of feature #1 - #6 of participants that underwent a MRI scan at two time points (i.e., in average of about 4.1 years apart). The proportions that remained the same between the time points stem from the identical participants. The differential score (DS) reflects the number of participants that expressed a change between the two time points. IPS = intraparietal sulcus, PoCS = postcentral sulcus, POS = paroccipital sulcus.

1. **Distribution of numerical abilities and sulcal pattern**


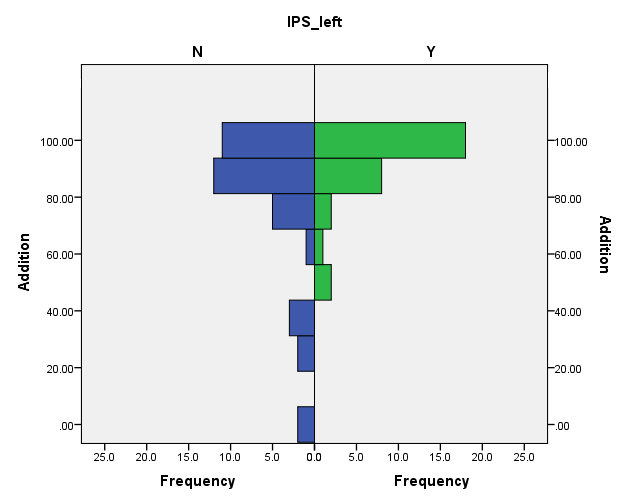

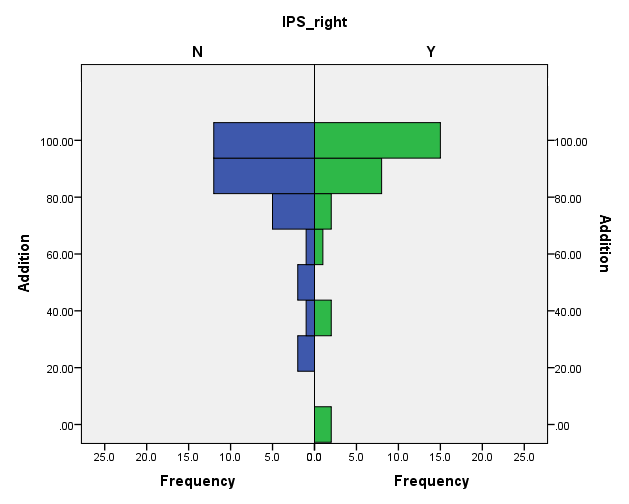

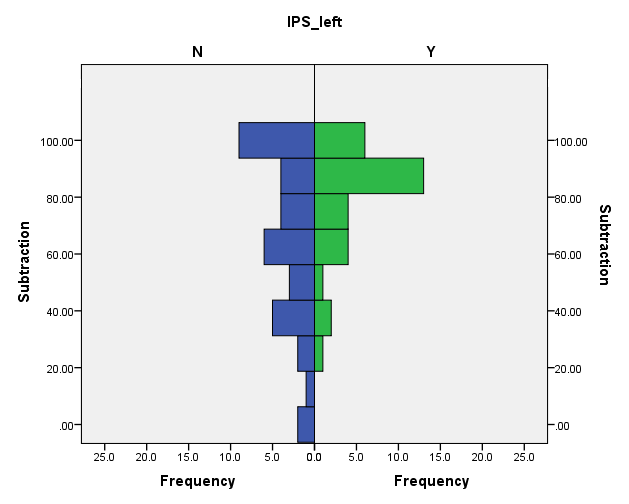

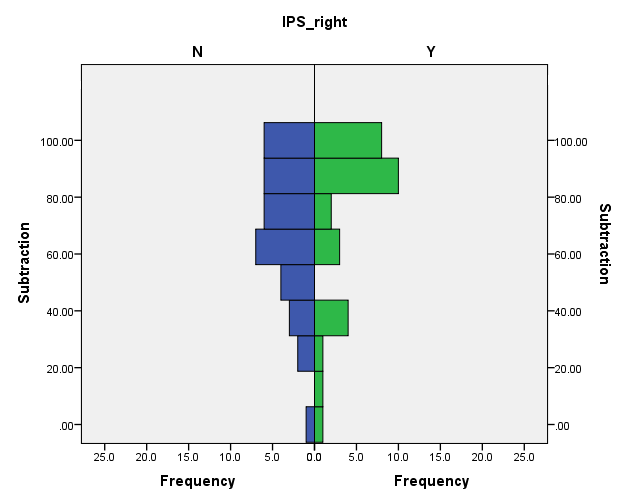

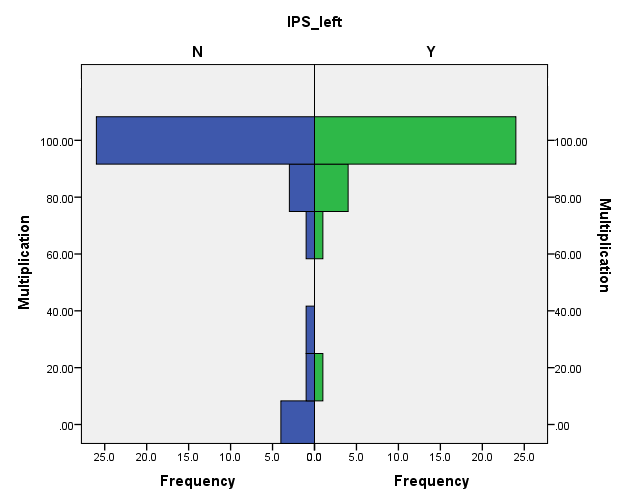

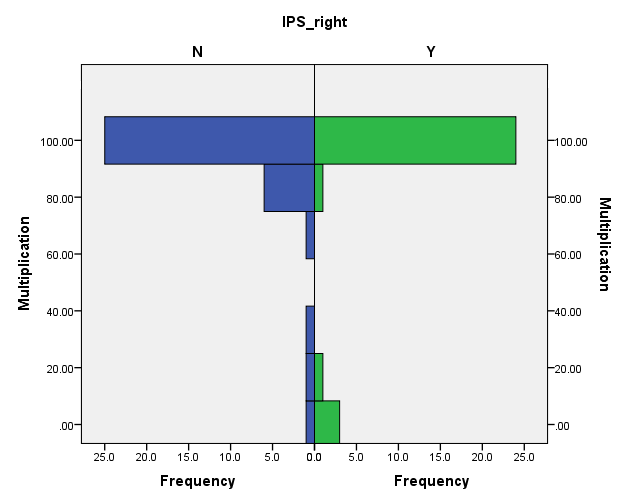

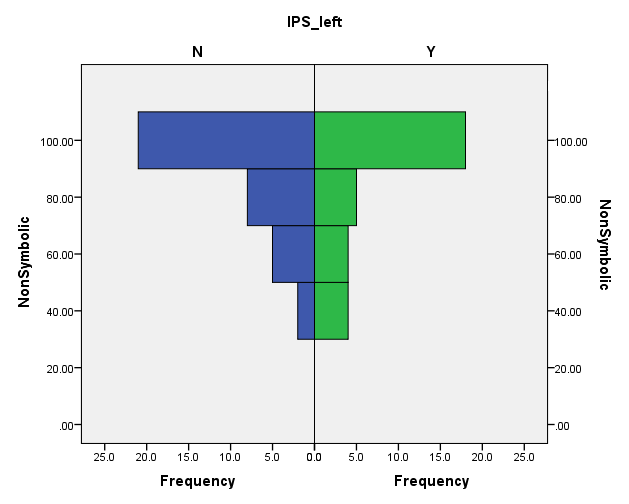

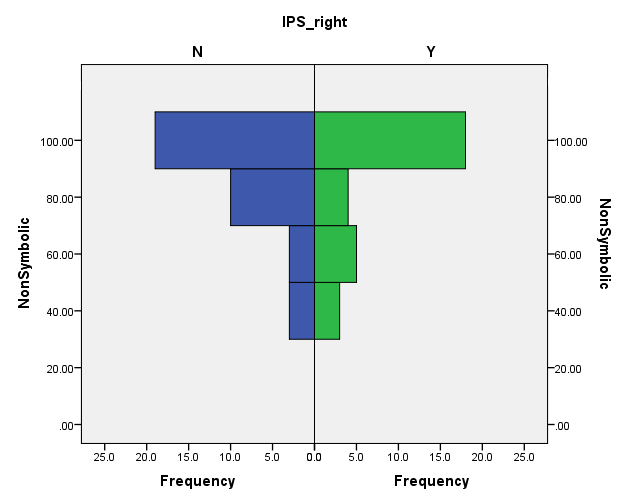

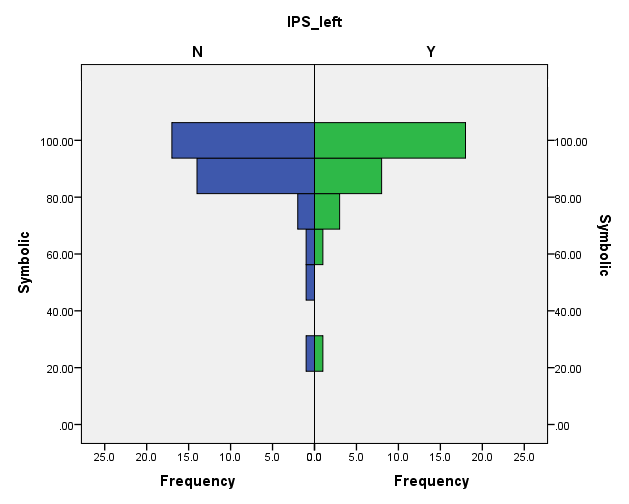

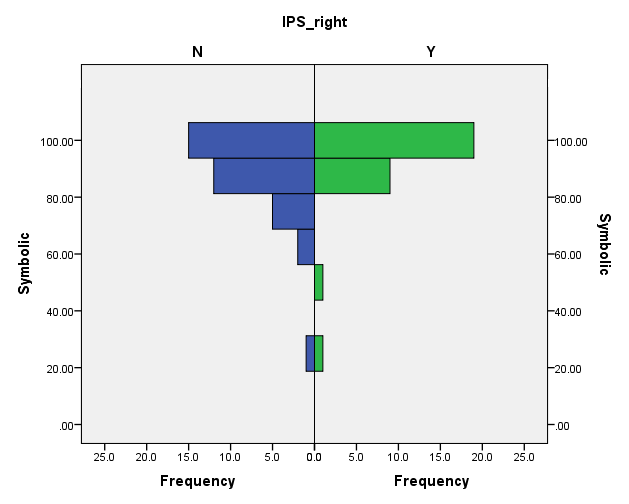


**Fig. 1d**

**Fig. 1c**

**Fig. 1a**

**Fig. 1b**

**Fig. 1f**

**Fig. 1e**

**Fig. 1h**

**Fig. 1g**

**Fig. 1j**

**Fig. 1i**

**Fig. 1a –j .** Distribution of percentage scores of the a priori analysed numerical abilities (i.e., 1a, 1b = addition, 1c, 1d = subtraction, 1e, 1f = multiplication, 1g, 1h = non symbolic quantity estimation, 1i, 1j = symbolic number comparison within sub-study 3 (N = 68, continous approach) divided for non-sectioned (N) and sectioned (Y) IPS for the left hemisphere on the left side and for the right hemisphere on the right side.

1. **Explorative analysis of diverse numerical abilities and IPS sulcal pattern**

Prior to conducting ANCOVA we tested whether our data of the numerical abilities we analyzed exploratory fulfilled the assumption of a significant correlation with age (see **Table 4**). See **Table 5a** for the mean and standard errors of the diverse numerical abilities and see **Table 5b** for the statistical results of the conducted ANCOVAs or ANOVAs.

**Table 4**. Correlation analyses between age and numerical abilities

|  |  | Age | |
| --- | --- | --- | --- |
|  |  | *r* | *p* |
| 1 | Enumeration | .148 | .228 |
| 2 | Counting backward | .296 | .014 |
| 3 | Writing numbers | .433 | < .001 |
| 5 | Reading numbers | .455 | < .001 |
| 6 | Number line | .247 | .042 |
| 7 | Digit span | .186 | .128 |
| 8 words | Oral number comparison | .233 | .056 |
| 10 | Contextual quantity estimation | -.030 | .808 |
| 11 | Story problems | .309 | .010 |

Note: Two-tailed Pearson's correlations (*r*) and p-values (*p*) for age and the remaining numerical abilities (ZAREKI-R subtests) used for the explorative analyses, degrees of freedom (df) for each of the correlations were 66.

**Table 5a**: Mean and Standard Errors of diverse numerical abilities (explorative analyses)

|  | Left IPS | | | | | | | |  | Right IPS | | | | | | | |
| --- | --- | --- | --- | --- | --- | --- | --- | --- | --- | --- | --- | --- | --- | --- | --- | --- | --- |
|  | Mean | | | | Standard Error | | | |  | Mean | | | | Standard Error | | | |
|  | Not Sectioned | | Sectioned | | Not Sectioned | | Sectioned | |  | Not Sectioned | | Sectioned | | Not Sectioned | | Sectioned | |
|  | F | M | F | M | F | M | F | M |  | F | M | F | M | F | M | F | M |
| Enumeration | 95.6 | 95.0 | 95.9 | 100 | 1.7 | 3.0 | 2.0 | 2.7 |  | 95.8 | 97.5 | 95.9 | 97.5 | 1.7 | 3.0 | 2.0 | 2.7 |
| Counting backwards | 82.1 | 89.5 | 86.2 | 91.4 | 5.7 | 9.9 | 6.4 | 8.8 |  | 81.2 | 97.7 | 87.1 | 83.3 | 5.7 | 10.0 | 6.5 | 8.8 |
| Writing numbers | 79.8 | 76.6 | 78.2 | 95.1 | 5.7 | 10.0 | 6.5 | 8.8 |  | 74.8 | 92.2 | 83.2 | 79.5 | 5.7 | 10.0 | 6.5 | 8.8 |
| Reading numbers | 84.3 | 89.1 | 85.2 | 100 | 4.5 | 7.8 | 5.1 | 6.9 |  | 84.3 | 93.6 | 85.2 | 95.3 | 4.5 | 7.8 | 5.1 | 6.9 |
| Number line | 73.9 | 82.3 | 76.4 | 82.2 | 3.5 | 6.2 | 4.0 | 5.5 |  | 75.4 | 78.4 | 74.9 | 86.1 | 3.6 | 6.3 | 4.0 | 5.5 |
| Digit span | 58.0 | 55.2 | 62.1 | 59.9 | 2.6 | 4.6 | 3.0 | 4.1 |  | 59.3 | 57.8 | 60.9 | 57.3 | 2.6 | 4.6 | 3.0 | 4.1 |
| Oral number comparison | 80.7 | 76.6 | 81.8 | 85.7 | 3.8 | 6.7 | 4.3 | 5.9 |  | 81.3 | 84.4 | 81.2 | 77.9 | 3.8 | 6.7 | 4.3 | 5.9 |
| Contextual quantity estimation | 88.0 | 100 | 83.7 | 85.1 | 3.7 | 6.5 | 4.2 | 5.7 |  | 84.7 | 87.5 | 87.0 | 97.6 | 3.67 | 6.4 | 4.2 | 5.7 |
| Story problems | 77.2 | 70.8 | 79.1 | 86.9 | 6.0 | 10.5 | 6.8 | 9.3 |  | 72.1 | 72.8 | 84.2 | 84.9 | 6.0 | 10.6 | 6.8 | 9.3 |

Note: F = female, M = male.

**Table 5b**. Results of ANOVAs or ANCOVAs of diverse numerical abilities (explorative analysis)

|  | Age | Left IPS | Right IPS | Interaction  left IPS x right IPS | |
| --- | --- | --- | --- | --- | --- |
|  |  |  |  |  |  |
| Enumeration |  |  |  |  |  |
| *F* | --- | 1.129 | 0.001 | 0.566 |  |
| *p* | --- | .292 | .977 | .455 |  |
| ηp² | --- | .020 | .000 | .010 |  |
|  |  |  |  |  |  |
| Counting backwards |  |  |  |  |  |
| *F* | 7.037 | 0.143 | 0.289 | 0.000 |  |
| *p* | .010 | .707 | .593 | .985 |  |
| ηp² | .113 | .003 | .005 | .000 |  |
|  |  |  |  |  |  |
| Writing numbers |  |  |  |  |  |
| *F* | 20.188 | 1.904 | 0.147 | 4.035 |  |
| *p* | .000 | .173 | .703 | .049 |  |
| ηp² | .269 | .033 | .003 | .068 |  |
|  |  |  |  |  |  |
| Reading numbers |  |  |  |  |  |
| *F* | 19.086 | 1.514 | 0.026 | 0.811 |  |
| *p* | .000 | .224 | .873 | .372 |  |
| ηp² | .258 | .027 | .000 | .015 |  |
|  |  |  |  |  |  |
| Number line |  |  |  |  |  |
| *F* | 4.82 | 0.06 | 0.537 | 1.611 |  |
| *p* | .032 | .808 | .467 | .210 |  |
| ηp² | .081 | .001 | .010 | .028 |  |
|  |  |  |  |  |  |
|  | Age | Left IPS | Right IPS | Interaction  left IPS x right IPS | |
| Digit span |  |  |  |  |  |
| *F* | --- | 1.409 | 0.021 | 0.112 |  |
| *p* | --- | .240 | .886 | .739 |  |
| ηp² | --- | .025 | .000 | .002 |  |
|  |  |  |  |  |  |
| Oral number comparison |  |  |  |  |  |
| *F* | --- | 0.937 | 0.391 | 1.212 |  |
| *p* | --- | .337 | .534 | .276 |  |
| ηp² | --- | .016 | .007 | .021 |  |
|  |  |  |  |  |  |
| Contextual quantity estimation | --- |  |  |  |  |
| *F* | --- | 3.503 | 1.478 | 4.535 |  |
| *p* | --- | .066 | .229 | .038 |  |
| ηp² | --- | .059 | .026 | .075 |  |
|  |  |  |  |  |  |
| Story problems |  |  |  |  |  |
| *F* | 6.536 | 1.161 | 2.125 | 0.488 |  |
| *p* | .013 | .286 | .151 | .488 |  |
| ηp² | .106 | .021 | .037 | .009 |  |

Note: *F* = F value, *p* = p-value, ηp² = partial eta squared, for all effects degrees of freedom (df) = 1, 56.

1. **Correlation analyses of IQ and sulcal pattern**

Although that all participants on individual and group level reached an IQ score in the normal range, TD significantly demonstrated higher IQs compared to DD. Therefore in order to evaluate whether IQ was significantly related to the sulcal pattern as well, we performed Spearman correlation analyses between the intelligence scores and the binary score of the of the sulcal pattern (i.e. sectioned IPS vs not sectioned IPS) of the entire sample of sub-study 2). This analysis revealed that the correlation was not significant, *rs* (84) = .045, *p* = .686.

1. **DD non-sectioned IPS subgroups: double horizontal IPS vs different IPS shape**

In a post hoc analysis we investigated whether participants with DD with a non-sectioned IPS in the left hemisphere do differ in terms of the presence of the double-horizontal shape compared (DD non-sectioned & double-horizontal) to those with a different IPS shape (non-sectioned & different) using t tests. Contrary to our expectations, DD double-horizontal demonstrated significantly higher accuracy scores in addition (DD non-sectioned & double-horizontal: *M* = 85.9, *SD* = 20.5; DD non-sectioned & different: *M* = 60.3, *SD* = 32.5, *t*(20.7) = -2.392, *p* = .026) and in subtraction (DD non-sectioned & double-horizontal: *M* = 68.8, *SD* = 24.1, DD non-sectioned & different: *M* = 44.1, *SD* = 26.2, *t*(23) = -.2.247 *p* = .035). However, both analyses did not pass the Bonferroni corrected threshold (*p* = 0.017). In multiplication the groups did not differ significantly (DD non-sectioned & double-horizontal: *M* = 85.4, *SD* = 24.3, DD non-sectioned & different: *M* = 68.6, *SD* = 44.0, *t*(22.2) = -.1.225, *p* = .234). Age did not differ significantly between the groups (DD non-sectioned & double-horizontal: *M* = 9.9, *SD* = 1.1, DD non-sectioned & different: *M* = 9.6, *SD* = 1.2, *t*(23) = 0.463, *p* =.551) which indicates that age was not significantly related to these results. In the right hemisphere, we did not find significant differences between the groups neither for addition (DD non-sectioned & double-horizontal: *M* = 67.2, *SD* = 28.3; DD non-sectioned & different: *M* = 79.2, *SD* = 21.5*, t*(18) = 1.076, *p* = .296), nor subtraction (DD non-sectioned & double-horizontal: *M* = 51.6, *SD* = 23.6, DD non-sectioned & different: *M* = 56.3, *SD* = 24.1, *t*(18) = .429, *p* = .673) nor multiplication (DD non-sectioned & double-horizontal: *M* = 87.5, *SD* = 23.1, DD non-sectioned & different: *M* = 79.2, *SD* = 34.9, *t*(18) = -.591, *p* = .562). Age did not differ between the groups (DD non-sectioned & double-horizontal: *M* = 9.9, *SD* = 1.1, DD non-sectioned & different: *M* = 9.7, *SD* = 0.9, *t*(18) = -.306, *p* =.763).

1. **DD subgroups: double horizontal IPS vs different IPS shape**

In a post hoc analysis we investigated whether participants with DD regardless whether they had a sectioned or non-sectioned IPS do differ in terms of their IPS shape such as presence of the double-horizontal IPS shape vs. another IPS shape. T-tests analyses revealed the following results. Left hemisphere: addition (DD double-horizontal: *M* = 84.7, *SD* = 19.5, DD different shape: *M* = 66.5, *SD* = 30.6, *t*(32) = -1.662, *p* = .11), subtraction (DD double-horizontal: *M* = 68.1, *SD* = 22.6, DD different shape: *M* = 48.5, *SD* = 25.9, *t*(32) = -2.006, *p* = .053) and multiplication (DD double-horizontal: *M* = 87.0, *SD* = 23.2, DD different shape; *M* = 72.2, *SD* = 40.4, *t*(25.2) = -1.309, *p* = .20). Age did not differ between the groups (DD double-horizontal: *M* = 10.1, *SD* = 1.1, DD different shape: *M* = 9.7, *SD* = 1.1, *t*(32) = -1.066, *p* = .30). Right hemisphere: addition (DD double-horizontal: *M* = 61.5, *SD* = 31.7, DD different shape: *M* = 73.7, *SD* = 27.3, t(29) = -1.141, *p* = .263), subtraction (DD double-horizontal: *M* = 47.9, *SD* = 23.7, DD different shape: *M* = 52.6, *SD* = 26.5, *t*(29) = -.501, *p* = .620) and multiplication (DD double-horizontal: *M* = 76.4, *SD* = 37.2, DD different shape: *M* = 75.0, *SD* = 40.1, *t*(29) = -.096, *p* = .925). Age did not differ between the groups (DD double-horizontal: *M* = 9.9 *SD* = 1.4, DD different shape: *M* = 9.7, *SD* = 0.9, *t*(29) = -.379, *p* = .708).

**10. Arithmetic accuracy and age in the sectioned left IPS group**


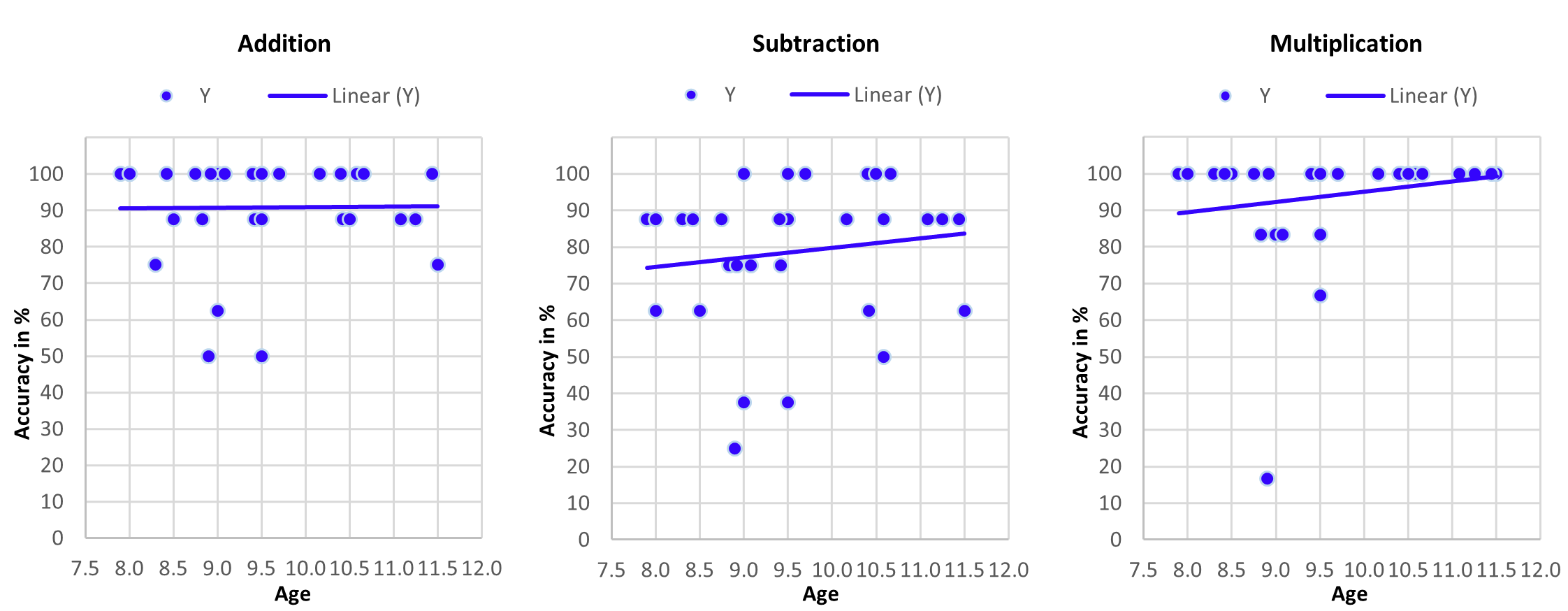


**Fig. 2**. Scatter plots illustrating statistically non-significant Kendall's tau correlations between age and the arithmetic accuracy scores in percentage (addition, subtraction and multiplication) for participants with a left sectioned IPS (n = 31) of the continuous sample of the third sub-study.

1. **References**

Faul, F., Erdfelder, E., Lang, A. G., & Buchner, A. (2007). G* Power 3: A flexible statistical power analysis program for the social, behavioral, and biomedical sciences. Behavior Research Methods, 39(2), 175-191.

Gaser, C., Dahnke, R., Thompson, P. M., Kurth, F., Luders, E., & Alzheimer’s Disease Neuroimaging Initiative. (2022). CAT–A computational anatomy toolbox for the analysis of structural MRI data. Biorxiv, 2022-06.

Roell, M., Cachia, A., Matejko, A. A., Houdé, O., Ansari, D., & Borst, G. (2021). Sulcation of the intraparietal sulcus is related to symbolic but not non-symbolic number skills. Developmental Cognitive Neuroscience, 51, 100998.
